# Hexane Fraction of *Turbo brunneus* Inhibits Intermediates of RANK-RANKL Signaling Pathway and Prevent Ovariectomy Induced Bone Loss

**DOI:** 10.3389/fendo.2019.00608

**Published:** 2019-09-06

**Authors:** Sachin Chaugule, Shalini Kashipathi Sureshbabu, Suresh Dakave, C. Murali Krishna, Pradip Chaudhari, Madhavi Indap, Shubhada Chiplunkar

**Affiliations:** ^1^Chiplunkar Lab, Advanced Centre for Treatment, Research and Education in Cancer, Tata Memorial Centre, Navi Mumbai, India; ^2^Central Research Laboratory, D. G. Ruparel College, Mumbai, India; ^3^Homi Bhabha National Institute, Mumbai, India; ^4^Chilakapati Laboratory, Advanced Centre for Treatment, Research and Education in Cancer, Tata Memorial Centre, Navi Mumbai, India; ^5^Comparative Oncology Program and Small Animal Imaging Facility, Advanced Centre for Treatment, Research and Education in Cancer, Tata Memorial Centre, Navi Mumbai, India

**Keywords:** *Turbo brunneus*, HxTME, osteoclastogenesis, RANKL, NFATc1, bone resorption, ovariectomy

## Abstract

Osteoporosis is a “silent disease” characterized by fragile and impaired bone quality. Bone fracture results in increased mortality and poor quality of life in aged people particularly in postmenopausal women. Bone is maintained through the delicate balance between osteoclast-mediated bone resorption and osteoblast-mediated bone formation. The imbalance is caused most often by overly active osteoclasts due to estrogen deficiency. Natural products have long been used to prevent and treat osteoporosis since they have fewer side effects. The marine environment is a potential source of biologically and structurally novel biomolecules with promising biological activities but is less explored for the treatment of bone-related diseases. The present study aims to evaluate the antiosteoporotic effect of Hexane fraction of *Turbo brunneus* methanolic extract (HxTME) and to investigate its role in RANK-RANKL signaling pathway using *in vitro* osteoclasts cultures and *in vivo* ovariectomized (OVX) Swiss mice model. The present study demonstrated that the HxTME significantly inhibited RANKL induced osteoclast differentiation and maturation *in vitro*. HxTME completely downregulated the mRNA expression of key transcription factors such as NFATc1, c-FOS, and osteoclasts related genes involved in osteoclastogenesis. *In vivo* studies also depicted the effectiveness of HxTME in ovariectomized mice by preserving bone microarchitecture, mineral content, and inhibiting bone loss in treated mice as analyzed by Histomorphometry, MicroCT, and Raman spectroscopy. Oral administration of HxTME fraction resulted in the decreased percentage of F4/80^+^, CD11b^+^, and CD4^+^ RANKL^+^ T cells in OVX mice whereas pro-osteoclastic cytokine, IL6 was markedly reduced upon treatment with HxTME. On stimulation with PMA/Io and PHA, a significant decrease in proliferative response in the splenocytes of HxTME treated OVX mice was observed. Fatty acid profiling revealed that HxTME is rich in ω3 and ω6 polyunsaturated fatty acids (PUFAs), which have high nutraceutical properties and are known to play important role in growth, development and maintenance of health. Therefore, HxTME may be a good source of nutraceutical in the treatment of bone-related diseases particularly in postmenopausal osteoporosis and may be pursued as a potential candidate for treatment and management of osteoporosis.

## Introduction

Bone is a dynamic tissue of the vertebrate body that not only provides mechanical support but also serves as a major storage site for minerals and growth factors. The integrity of bone is maintained through a bone remodeling process which is characterized by a delicate balance between osteoclast-mediated bone resorption and osteoblasts mediated bone formation (1). The progressive imbalance between bone formation and bone resorption processes is manifested in several pathological conditions such as osteoporosis, Paget's disease, and Rheumatoid Arthritis (2, 3). Osteoporosis is the most common bone related disease in the aged community due to reduced bone mass and micro-architectural disintegration with increased bone fractures induced by hormonal imbalance (4).

Estrogen deficiency in menopausal women leads to the common form of osteoporosis known as postmenopausal osteoporosis. The decline in estrogen level not only affects osteoclastogenesis and osteoblastogenesis but also regulates the activity of T cells (5, 6). Receptor activator of the nuclear factor kappa B Ligand (RANKL) and Macrophage- Colony Stimulating Factor (M-CSF) are essential key factors produced by T cells and osteoblasts that promote osteoclasts proliferation and differentiation. The activation and formation of mature osteoclasts are achieved through binding of RANKL to its cognate receptor RANK present on the cell surface of osteoclast precursors (7). Binding of RANK-RANKL triggers trimerization of TNF receptor-associated factor 6 (TRAF6) in osteoclast precursor cells which activates downstream signaling pathways such as p38 mitogen-activated protein kinase (MAPK), c-Jun-N-terminal kinase (JNK), extracellular signal-regulated kinase (ERK), and NF-kB. Activation of MAPKs and NF-kB consequently results in expression of key transcriptional factors such as c-Fos and NFATc1 which are essential for osteoclasts differentiation (8). Particularly, NFATc1 is a master regulator of osteoclast differentiation. It regulates a number of osteoclast-specific genes that include cathepsin k, Tartrate-resistant acid phosphatase (TRAP), osteoclast-associated receptor (OSCAR), calcitonin receptor, and matrix metalloproteinase's (MMPs) (9). However, the crucial role of c-Fos in osteoclast differentiation has been established by the observation that the c-Fos^−/−^ mice develop osteopetrosis owing to impairment of osteoclastic activity (10). Due to the pivotal role of these transcription factors and signaling molecules in osteoclastogenesis, it is important to regulate their activation and expression in the treatment of bone-related diseases.

Currently, estrogen treatment or hormone replacement therapy, antiresorptive agents like bisphosphonates and anabolic agents like Parathyroid Hormone (PTH) have emerged as potential therapeutics for osteoporosis treatment. All these formulations offer much promise but the long-term safety of these treatments remains questionable due to their side effects (11). Therefore, there is a need to identify efficacious antiresorptive agents which are safe. Marine natural products are gaining attention as attractive and safe resources for therapeutic application and drug discovery. Marine animals produce an overabundance of structurally distinct and biologically active secondary metabolites which can be explored for the discovery of novel drugs (11, 12). Recently, mactanamide, a new fungistatic diketopiperazine isolated from the marine fungus *Aspergillus flocculosus* derived from a sponge *Stylissa* sp. displayed a potent suppressive effect on osteoclast differentiation without any cytotoxicity (13). Earlier work from our group has shown that a mollusk *Turbo brunneus* can inhibit bone resorption by regulating T cell activity (6). Based on this background, we have further investigated the anti-osteoclastogenic effect of Hexane fraction derived from the crude methanolic extract of *Turbo brunneus* (TME) and its mechanism of action in osteoclast related RANK-RANKL pathway. In this study, we have shown that the hexane fraction of *Turbo brunneus* (HxTME) inhibits osteoclast differentiation and maturation *in vitro*. It directly inhibits the pro-osteoclastic stimulus provided by T cells. It was also noted that a marked increase in T cell proliferation owing to estrogen deficiency was significantly attenuated by HxTME supplementation. *In vivo* studies revealed that the HxTME preserves bone microarchitecture through maintaining bone mineral density (BMD), connectivity index, trabecular networking, and mineral content in bilaterally ovariectomized mice. Fatty acid profiling revealed that HxTME is rich in ω3 and ω6 PUFAs, with a healthier ratio of ω6/ω3.

## Materials and Methods

### Reagents and Mice

Recombinant mouse M-CSF and recombinant mouse RANKL were purchased from R&D Systems, USA. Primary antibodies to c-Jun, Akt, MAPK p38, ERK 1/2, phospho-ERK 1/2, IKK β, and phospho-IKK β were obtained from Cell Signaling Technology (CST), Danvers, MA. Primary antibody to β-actin, anti-mouse, and anti-rabbit HRP conjugated secondary antibodies were purchased from Sigma-Aldrich, USA. Female Swiss albino mice (18–22 g) were procured and maintained at the animal house facility of ACTREC. All *in vivo* protocols and experiments were carried out in accordance with the relevant guidelines approved by the Institutional Animal Ethics Committee, ACTREC. Animals were fed with standard laboratory diet and water *ad libitum*.

### Extraction, Fractionation, and Preparation of HxTME Fraction

Procedure for extraction of a crude methanolic TME was described previously (6). The crude methanolic extract was further fractionated by liquid-liquid chromatography or partition chromatography which depends on the distribution of each component of a mixture between two immiscible liquids (14). The crude methanolic TME was successfully partitioned with Hexane (1:1 v/v). This solvent mixture was thoroughly equilibrated in a separating funnel at room temperature and was separated into two phases i.e., methanol soluble fraction and a hexane soluble fraction. This hexane soluble fraction was labeled as HxTME. This fraction was dried and stored at −20°C. HxTME was solubilized in DMSO before use for *in vitro* and *in vivo* studies.

### Cell Culture and *in vitro* Osteoclastogenesis

Mouse BMMs were prepared with minor modification as described previously by Kim et al. (15) and Bradley and Oursler (16). For primary cell culture, Bone marrow cells were obtained by flushing bone marrow cavity of tibiae and femora of 5–6-week-old female Swiss mice with α-Minimum essential medium (α-MEM, Sigma-Aldrich, USA). Bone marrow cells were cultured overnight in α-MEM medium containing 10% Fetal Bovine Serum (FBS), 2 mM L-glutamine, and 1% penicillin-streptomycin (PenStrep) antibiotic solution supplemented with M-CSF (10 ng/ml). After 24 h, non-adherent bone marrow cells as osteoclast precursors were separated and further incubated in complete α-MEM with M-CSF (30 ng/ml) for 3 days. Floating cells were discarded followed a gentle PBS wash. Now, the Adherent cells were referred as Bone Marrow Macrophage cells (BMMs) (15). The adherent BMMs were harvested by trypsinization and used for further experiments. For osteoclast differentiation, BMMs (1 × 10^5^ cells/well) were cultured in complete α-MEM medium supplemented with RANKL (30 ng/ml) and M-CSF (30 ng/ml) for 7–8 days in a 96-well plate. Various concentrations of HxTME were added to cultures simultaneously at 0 day and cultures were replenished with fresh medium and treatments after every 3 days. After culturing for 7–8 days, cells were fixed for TRAP staining using TRAP staining kit (Sigma-Aldrich). Cells were then washed with distilled water and air dried for imaging and counting. TRAP-positive multinucleated cells containing more than three nuclei were considered and counted as mature osteoclasts.

### Bone Resorption Assay and F-actin Ring Formation

Bone Marrow Macrophage cells (0.1 × 10^6^/200 μl) were cultured in complete α-MEM medium containing 10% FBS and 1% penicillin-streptomycin (PenStrep) supplemented with M-CSF (30 ng/ml) and RANKL (30 ng/ml) in presence of different concentrations of HxTME or vehicle control (DMSO) into 96 well Corning Osteo Assay plate (Corning Cat. No. 3988) to begin the differentiation process. Negative control wells received 100 μl of medium (without cells) for future staining and pit visualization. Plates were incubated at 37°C in a humidified atmosphere of 5% CO_2_ for 7–8 days with intermediate media and supplement change. To analyse the surface for pit formation, on 7–8 day of culture cells were bleached using a 10% bleach solution. The wells were air dried and individual pits or multiple pit clusters were imaged using an inverted microscope at 100x magnification. The resorptive ability of osteoclasts was assessed by quantifying the total resorbed area using ImageJ Software.

For F-actin ring formation, briefly, BMM cells were cultured on Thermonox coverslips with M-CSF (30 ng/ml) and RANKL (30 ng/ml) with presence of different concentrations of HxTME fraction for 7–8 days. Further, the cells were washed with 1XPBS and fixed in 4% paraformaldehyde for 15 min at 4°C. Then, cells were stained with Phalloidin-TRITC for 30 min. Following incubation, the cells were washed and stained for nuclear dye DAPI (10 ng/ml). Phalloidin-stained cells were imaged using LSM 510 microscope and images were analyzed on LSM Image Analyzer.

### Western Blot Analysis

For Western blotting, BMMs were pre-treated with HxTME (25 μg/ml) for 3 h in the presence of M-CSF (30 ng/ml) and then stimulated with RANKL (30 ng/ml) for the indicated times (17). After every time point, whole cell lysates were prepared using NP-40 (Nonidet-P) lysis buffer along with a protease inhibitor cocktail (PIC) and phosphatase inhibitor. Total protein concentration was quantified by Bradford's assay. Equal amounts of protein were resolved on 8–10% SDS-PAGE gels and then transferred to Hybond-ECL nitrocellulose membrane (Amersham Pharmacia Biotech, Piscataway, NJ). The membranes were further blocked by using 5% bovine serum albumin for 1 h to avoid non-specific interaction and then membranes were probed with the specific primary antibodies for overnight at 4°C. After washing with Tris-buffered saline containing 0.1% Tween-20 (TBST), then the membranes were further probed with the appropriate HRP conjugated secondary antibodies. The specific signals were visualized with an ECL plus Western blot detection system (Amersham Pharmacia).

### Quantitative Real-Time PCR

Total RNA was extracted from BMM cells treated with different concentrations of HxTME at two different time points (3 and 7 days) using TRIzol reagent (Invitrogen Life Technologies, NY, USA) and reverse transcribed to cDNA using superscript cDNA synthesis kit (Invitrogen Life Technologies, NY, USA) as per manufactures instructions. The cDNA was used to quantitate different target genes using RT-PCR. Quantitative RT-PCR for different genes was performed with PRISM 7700 (PE Applied Biosystems, Foster City, CA). Samples were analyzed using TaqMan primer sets purchased from Applied Biosystems: NFATc, Mm00479445_m1; Fos, Mm00487425_m1; Cathepsin K, Mm00484036_m1; Trap, Mm00446003_m1; and 18sRNA, Mm03928990_g1. All values were normalized to the expression of the housekeeping gene 18s RNA. Relative gene expression was quantified by using Applied Biosystems real time PCR system software.

### Intracellular ${\text{Ca}}_{2}^{+}$ Staining

Mouse BMM cells were (1 × 10^6^/ml) were incubated with M-CSF (30 ng/ml) and RANKL (30 ng/ml) in presence and absence of HxTME for 24 and 48 h. Cells were then incubated for 30 min in presence of 5 μm Fluo 3 AM and 0.05% Pluronic F127 in ${\text{Ca}}_{2}^{+}$ estimation buffer (137 mM NaCl, 5 mM KCl, 1 mM Na_2_HPO_4_, 10 mM Glucose, 1 mM MgCl_2_, 20 mM Hepes, 0.1% BSA, 1mM CaCl_2_). After incubation, cells were washed with calcium buffer and centrifuged at 1,500 rpm for 10 min. Finally, the cells re-suspend in 500 μl of calcium estimation buffer. Cells were excited at 480 nm and emission at 505–530 nm was acquired on FACS Aria and also on a confocal microscope. Mean Fluorescence Intensity (MFI) was measured using FlowJo software.

### *In vivo* Animals Studies

This study was carried out in accordance with the principles of the Basel Declaration and recommendations of Institutional Animal Ethics Committee (IAEC), Advanced Center for Treatment, Research and Education in Cancer (ACTREC) which is endorsed by the Committee for the Purpose of Control and Supervision of Experiments on Animals (CPCSEA), Ministry of Environment, Forest and Climate Change, Govt. of India. The protocol was approved by the Institutional Animal Ethics committee (IAEC, Project No. 03/2014), ACTREC. Swiss Albino mice were housed in cages (Temperature 25 ± 2°C) with 12 h light and dark cycle and fed on standard lab diet *ad libitum*. Following 1 week's acclimatization, the female mice were subjected to bilateral ovariectomy (OVX) and Sham surgery. The mice were randomly divided into seven groups consisting of five animals in each group as follows: Group A: bilaterally ovariectomized control mice treated with vehicle (OVX); Group B: Sham control mice treated with vehicle (Sham control); Group C: OVX mice treated with estradiol-2 mg/kg body weight (positive control); Group D: OVX treated with 25 mg/kg body weight; Group E: OVX treated with 12.5 mg/kg body weight; Group F: OVX mice treated with 6.25 mg/kg body weight; and Group G: OVX mice treated with 3.125 mg/kg body weight. After 7 days of recovery from surgical convalescence, vehicle, estradiol, and HxTME were administered orally through oral gavages to all animal groups, respectively. We used estradiol as the positive control because bone conserving effects of estrogens are well-established in the ovariectomized mice model of osteoporosis (18). The body weight of the animals was recorded weekly during the experimental period. At the end of 5 weeks, mice were euthanized by carbon dioxide asphyxiation. Spleen, Uteri, Femora, and tibiae were harvested. Blood samples were collected and centrifuged for serum isolation and stored at −80°C for biochemical analysis.

### MicroCT of Bones

Femurs were scanned using the skyscan 1174 compact desktop microCT (Bruker microCT, Kontich, Belgium). Each sample was carefully positioned such that the orientation of the femoral shaft was as vertical as possible. Femurs were scanned at a focal spot of 84 μM by applying the following settings: X-ray voltage 40 kV, electric current 790 μA, Exposure 600 ms, FOV (field of view) 29.3 mm, and 4X magnification. Morphologic measurements of the proximal trabecular bone were performed and analyzed using MicroView v. 2.0 Software. Following 3D parameters obtained are BMD, bone volume (BV)/tissue volume (TV), (BV/TV), trabecular thickness (Tb. Th), trabecular separation (Tb. Sp), and connectivity index. For histological analysis, bones and uterus were fixed with Bouin's solution for 2 h, which was followed by decalcification in 5% nitric acid for bones, dehydration in alcohol, clearing in xylene and finally embedding in paraffin for further tissue sections. Histomorphometric analysis was done on an upright microscope after hematoxylin and eosin (H&E) staining.

### Raman Spectroscopy of Bones

Raman spectra were recorded from the shaft-neck region of the bones obtained from OVX, Sham, Estradiol and HxTME treated groups of mice as described earlier (6). The spectra were recorded at different points with spacing of 1 mm and were acquired using Raman microscope WITec alpha300RS (WITec GmbH, Ulm, Germany). The instrument was calibrated for spectra using argon mercury lamp. For excitation at 28 mW, a 532 nm Nd:YAG laser was focused by a 10X (0.25NA, Zeiss) objective onto the sample and resultant Raman signals were detected using 300 nm spectrograph with 600 g/mm grating coupled with a charged couple device (CCD). Spectra were integrated for 5 s and averaged over 10 accumulations. Raman spectra were acquired in the 800–1,800 cm^−1^ region with an integration time of 5 s/spectrum. Parameters were kept constant for all measurements. On an average nine spectra were recorded for each bone from Group A–G corresponding to total 258 spectra. Spectra were corrected for background signals and normalized. Band fitting was done using a Gaussian function and fitting was undertaken until reproducible and converged results were obtained with squared correlations better than *r*^2^ −0.9998. The Area under the 960 and 1,650 cm^−1^ band was calculated using a band fitting algorithm of GRAMS/AI 7.02 (Thermo Electron Corporation) software.

### Bone Resorption Markers and Cytokine Analysis

Before animal euthanization, blood was collected and serum was separated and stored at −80°C. Serum Calcium and Acid phosphatase activity (ACP) were estimated spectrophotometrically as per the manufacturer's instructions (Span Diagnostic Kit, Span Diagnostic Ltd., India). IL6 was determined by using mouse Cytometric Bead Array kit (BD Biosciences, USA) as per the kit instructions. Samples were acquired on FACS Aria cytometer (BD Bioscience, San Jose, CA, USA). The data was analyzed using FCAP Array software (version 1.0, BD Biosciences, USA).

### Isolation and Immunophenotyping of Lymphocytes From Spleen

Splenocytes were isolated under aseptic conditions from all mice groups and layered on Ficoll Hypaque for density gradient separation of the splenic lymphocytes. These splenic lymphocytes were stained for various cell surface and intracellular markers such as CD3, CD4, CD8, CD19, CD11b, F4/80, and RANKL. Briefly, Aliquots of 1 × 10^6^ splenic lymphocytes were washed with ice-cold 1XPBS and fixed with 1% paraformaldehyde for 15 min at 4°C. The cells were washed with FACS buffer (1XPBS + 2% FBS + 0.001% sodium azide) and then labeled with fluorophore tagged antibodies CD3-PB, CD4-PECF594, CD8-PE, CD19-FITC, CD11b-Percp cy5.5 (BD Biosciences, USA), and F4/80 APC-cy7, RANKL-PE (BioLegend, San Diego, USA). Further, the cells were washed with FACS buffer and acquired on FACS Aria (BD Biosciences San Jose, USA). Data analysis was done using FlowJo software (Tree Star, Ashland, USA).

### T cell Proliferation Assay

The proliferation of splenic lymphocytes was determined by the ^3^H Thymidine incorporation assay. Lymphocytes were cultured at a density of 1.5 × 10^5^ cells/well in 96 well microtiter plates. Lymphocytes were stimulated with 1% Phytohaemagglutinin (PHA), and 50 ng/ml Phorbol 12-myristate 13-acetate (PMA) with 1 μg/ml Ionomycin (Sigma-Aldrich, St. Louis, MO, USA) in complete medium (RPMI + 10%FBS) and incubated in a humidified atmosphere of 5% CO_2_ at 37°C for 48 h. After 48 h, ^3^H Thymidine (0.5 μCi/10 ul/well) was added and the cells were further incubated for a period of 18 h. The radioactivity incorporated into DNA was measured using liquid scintillation counter (Packard TRI-CARB 2100 TR counters; Downers Grove, IL, USA) and data expressed as count per minute (CPM).

### GCMS Analysis of HxTME

The fatty acid content of the extract was determined by GCMS analysis. The fatty acids were analyzed by GC (Shimadzu GC-2010) fitted with a flame ionization detector (FID). The initial temperature of the oven was 50°C which was raised to 230°C at the rate of 4°C/min, and held at 230°C, while both injector and detector temperature were set at 250°C. The sample size was 1 μl. The fatty acid methyl esters separated by GC were further analyzed by MS measurements carried out in a Shimadzu QP2010 mass spectrophotometer with ionization energy of 70 eV. The mass spectrophotometer was tuned to get the relative abundance of m/z ranging from 40.00 to 400.00. Chromatographic data were recorded, and peaks were identified by comparing with FAME standards.

### Statistical Analysis

Data were presented as mean ± SEM and as mean ± SD wherever applicable. Statistical analysis was performed using Prism 6 (GraphPad Prism software). Statistical differences were assessed by Student *t*-test or one-way ANOVA where appropriate. Levels of *p* values of < 0.05, 0.01, and 0.001 were considered statistically significant.

## Results

### HxTME Fraction Inhibits RANKL-induced Osteoclastogenesis

**Figure 1:**
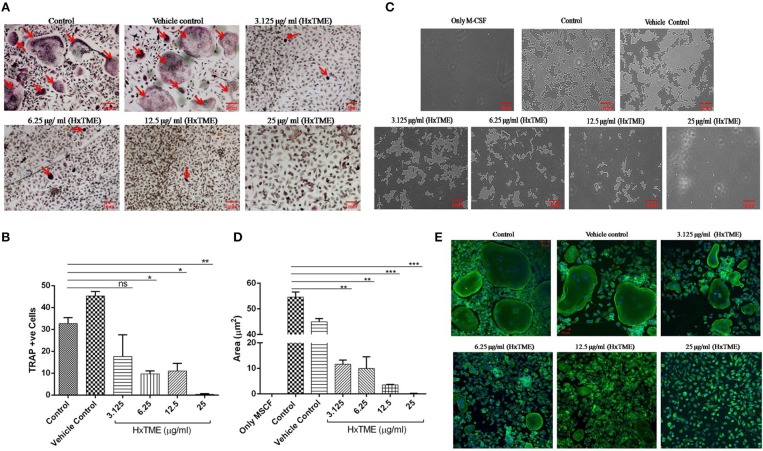
HxTME fraction inhibits RANKL-induced osteoclastogenesis, bone resorption, and F-actin ring formation. BMM cells were cultured with M-CSF (30 ng/ml) and RANKL (30 ng/ml) in presence of indicated concentration of HxTME. **(A)** Representative images of TRAP stained cells after 7–8 days. **(B)** Quantitative analysis of mature osteoclasts studied by counting multinuclear TRAP +ve osteoclasts with more than three nuclei. **(C)** BMM cells were cultured on calcium carbonate coated Osteo assay plate with M-CSF (30 ng/ml) and RANKL (30 ng/ml) to differentiate into osteoclasts, in the presence of HxTME. After 7–8 days incubation, cells were lysed and pit formation was observed under upright microscopy (magnification, 5X). Representative images of resorption pit formed after the fraction treatment for 7–8 days of incubation. **(D)** Representative results are expressed in resorptive pit area/well (μm^2^) from three independent experiments. **(E)** BMM cells were cultured in medium with M-CSF (30 ng/ml) and RANKL (30 ng/ml) to differentiate into osteoclasts, in the presence of HxTME. After 7–8 days incubation, cells were labeled with Alexa 488-conjugated phalloidin (green) and DAPI (blue). Representative images of actin ring formation were taken under a confocal microscope at a magnification of 5X. All values were expressed as Mean ± SEM of three independent experiments. Scale bar = 1mm, ns = non-significant, ^*^P ≤ 0.05, ^**^P ≤ 0.01, ^***^P ≤ 0.001 vs. M-CSF and RANKL treated Control (Control).

In the present study, we examined the inhibitory effects of HxTME on RANKL-induced osteoclastogenesis in the BMM cells. The BMM cells were cultured in α MEM medium with various concentrations of HxTME fraction ranging from 3.125 to 25 μg/ml in presence of M-CSF and RANKL for 7–8 days. Under normal control conditions, RANKL treated BMM cells differentiated into multinucleated giant cells through repetitive cell fusion. TRAP staining confirmed the presence of multinucleated TRAP+ve osteoclasts in wells treated with RANKL and M-CSF. BMM cells treated with HxTME significantly decreased the number of TRAP+ve osteoclasts and also caused a dramatic change in their morphology at all concentration (3.125–25 μg/ml) as compared to the control cells (Figures 1A,B). To rule out the toxicity associated with HxTME, MTT assay was carried out. The results demonstrated that HxTME fraction does not affect the viability of BMM cells (Data not shown).

### HxTME Inhibits Bone Resorption and F-actin Ring Formation

Polarization is the most initial step in bone resorption which is marked by the actin-ring organization at the periphery of osteoclasts. Since osteoclasts form pit after bone matrix resorption, it is possible to measure the functional bone resorption activity of osteoclasts. Area of resorbed pits was measured using upright microscopy and the phalloidin-labeled actin rings can be recognized as bright red/green belts at the periphery of osteoclasts (Figures 1C–E). HxTME significantly suppressed the pit forming capacity of RANKL induced BMM cells at all concentrations. HxTME treatment significantly reduced total resorption area at all concentrations as compared to the control. Subsequently, the effect of HxTME was also examined on F-actin ring formation as shown in Figure 1E. The presence of HxTME led to a marked reduction in the size and distortion in F-actin ring formation in a dose dependent manner (Figure 1E). Altogether, these results suggest that HxTME suppressed not only RANKL-induced osteoclastogenesis but also bone resorption through inhibiting pit formation and F-actin ring formation.

### HxTME Downregulates the RANKL-induced Expression of NFATc1, c-Fos, and Osteoclast Marker Genes

**Figure 2:**
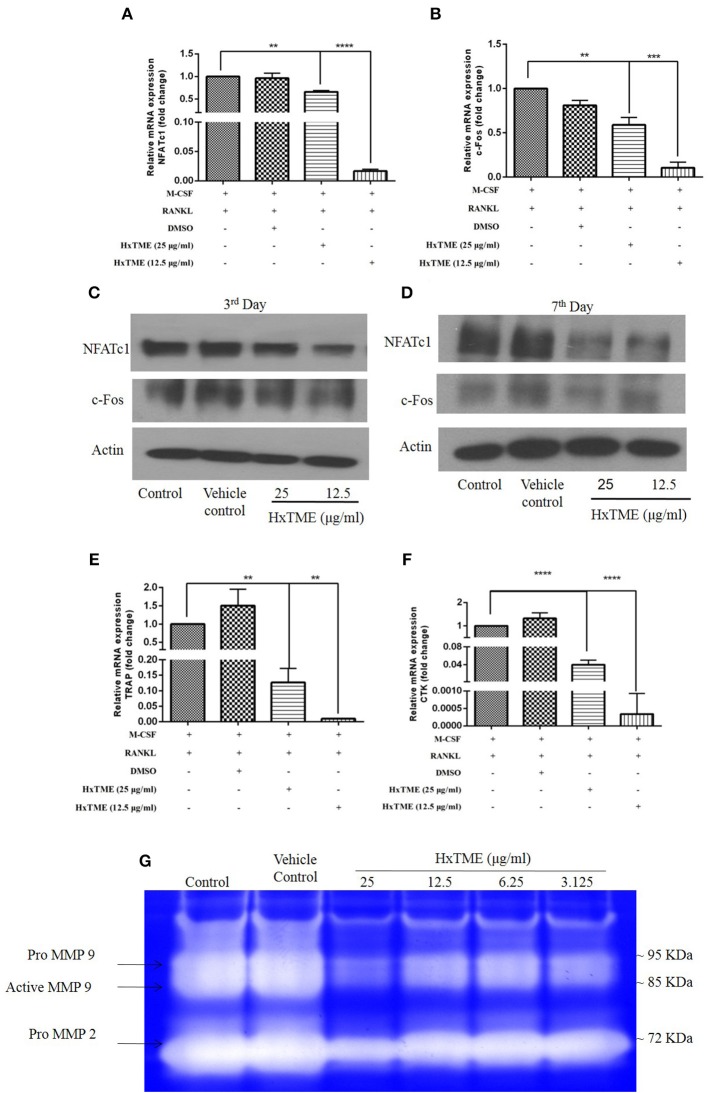
HxTME suppresses expression of NFATc1/c-Fos and osteoclast marker genes. BMM cells were cultured with M-CSF (30 ng/ml) and RANKL (30 ng/ml) in presence of indicated concentration of HxTME for 7 Days. Real-Time PCR was performed on RNA extracted from cells stimulated with M-CSF and RANKL in addition to indicated concentrations of HxTME. Gene expression of NFATc1, c-Fos, TRAP, and Cathepsin K was normalized to 18s RNA. The results indicated are cumulative mean of relative gene expression compared with RANKL control. **(A)** mRNA expressions of NFATc1 at 7th day. **(B)** mRNA expressions of c-FOS at 7th day. **(C,D)** BMM cells were cultured with M-CSF (30 ng/ml) and RANKL (30 ng/ml) in presence of HxTME (12.5 and 25 μg/ml) for 3 and 7 days. The cell lysates were analyzed for protein expression of c-Fos, NFATc1, and β-actin by western blotting. **(E)** mRNA expressions of TRAP at 7th day. **(F)** mRNA expressions of Cathepsin k at 7th day. All values were expressed as Mean ± SEM of three independent experiments. ^**^P ≤ 0.01, ^***^P ≤ 0.001, and ^****^P ≤ 0.0001 vs. M-CSF and RANKL treated control. **(G)** Representative image of gelatin zymogram showing MMP2 and MMP9 activities in conditioned medium of BMM cells cultured with different concentrations of HxTME fraction in presence of M-CSF (30 ng/ml) and RANKL (30 ng/ml).

To understand the mechanism of action by which HxTME fraction exerts its inhibitory role on the differentiation of BMM cells into mature osteoclasts, we examined the influence of HxTME on different key molecules engaged in the RANK-RANKL signaling pathway. Mice BMM cells were cultured with various concentrations of HxTME in presence of M-CSF (30 ng/ml) and RANKL (30 ng/ml) for 7 days' time period and further, mRNA expression of NFATc1, c-Fos, and osteoclast marker genes (TRAP and Cathepsin K) were measured using Real Time PCR. Our data demonstrated that there was a significant inhibition of mRNA expression of NFATc1 observed at a concentration of 25 μg/ml (*p* < 0.01) and 12.5 μg/ml (*P* < 0.0001) of HxTME (Figure 2A) on 7th day compared to control. However, mRNA expression of c-Fos was also found to be suppressed at a concentration of 25 μg/ml (*p* < 0.01) and 12.5 μg/ml (*P* < 0.0001) of HxTME on 7th day as compared to control (Figure 2B). Since, NFATc1 and c-Fos play essential role in osteoclast differentiation and are known to be upregulated during osteoclastogenesis, we investigated the effect of HxTME on expression of NFATc1 and c-Fos at protein level. We observed that the protein expression of transcription factors, NFATc1 and c-Fos was increased upon treatment with RANKL and M-CSF. The RANKL induced increased expression of NFATc1 and c-Fos was significantly reduced by HxTME throughout the experimental period (3rd and 7th day) (Figures 2C,D).

RANK-RANKL binding upregulates specific genes associated with osteoclast differentiation (8). We therefore investigated the expression of osteoclast related genes such as TRAP and Cathepsin K in mouse BMM cells (Figures 2E,F). The mRNA levels of TRAP and Cathepsin k was significantly reduced at concentration of 25 μg/ml (*p* < 0.01) and 12.5 μg/ml (*p* < 0.0001) of HxTME as compared to the RANKL and M-CSF stimulated control (Figures 2E,F). Our data suggest that HxTME treatment significantly decreased the RANKL induced mRNA expression of transcription factors (NFATc1, c-Fos) and osteoclast related genes such as TRAP and cathepsin K in BMM cells.

Matrix metalloproteinase (MMPs) are a group of proteolytic enzymes involved in the degradation of the extracellular matrix including bone. MMP2 and MMP9 produced by osteoclast and osteoblasts are critically involved in bone resorption. Expression of MMP2 and MMP9 in cells-free conditioned medium of osteoclasts cultures was examined by gelatin zymography (Figure 2G). The activity of MMP2 and MMP9 was markedly suppressed by HxTME at all concentrations ranging from 3.125 to 25 μg/ml. Gelatinolytic activity of MMP2 and MMP9 by mature BMM cells was suppressed by addition of HxTME fraction. The downregulation of MMP2 and MMP9 proteolytic activity might also be contributing to the anti-osteoporotic effect of HxTME.

### Effect of HxTME on RANKL-induced Intracellular Signaling Intermediates

**Figure 3:**
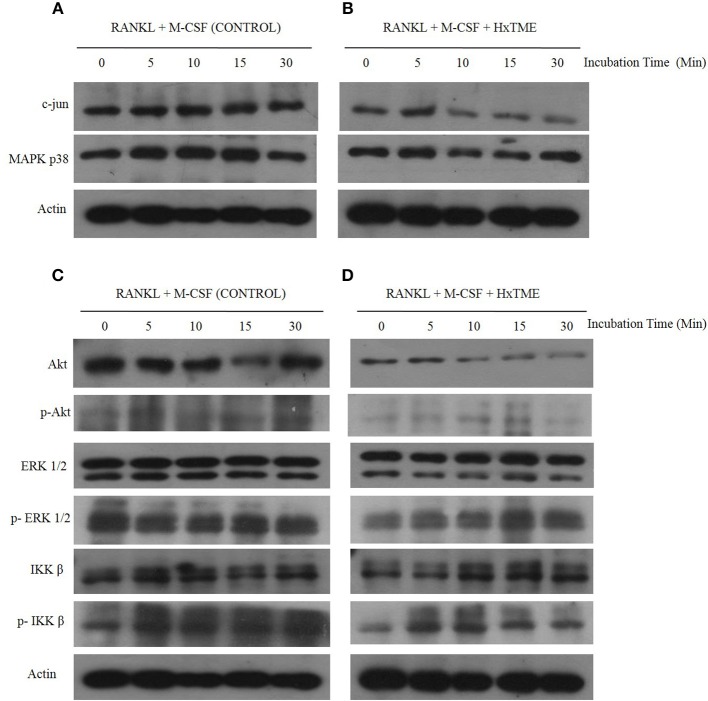
HxTME inhibits RANKL-induced c-jun, Akt, MAPK p38, and ERK1/2 activation. BMM cells were pre-treated with HxTME (25 μg/ml) and M-CSF (30 ng/ml) for 3 h and stimulated with RANKL (30 ng/ml) for the indicated times. Total cell lysates were prepared at the indicated time points and then subjected to Western blot analysis using specific antibodies as shown in **(A,B)**. Protein expression levels of c-jun, and MAPK p38 at indicated time points in BMM cells stimulated with only RANKL and M-CSF (Control) and with RANKL, M-CSF, and HxTME. **(C,D)** Protein expression of Akt, p-Akt, ERK, p-ERK, IKKβ, and p-IKKβ at indicated time points in BMM cells stimulated with only RANKL and M-CSF (Control) and with RANKL, M-CSF, and HxTME. All samples were run together with the same loading control, β-actin. Loading control, β-actin was re-used for illustrative purposes in **(A–D)**.

During osteoclastogenesis, RANKL stimulation activates various intracellular signaling cascades. To understand the signaling pathways mediated by HxTME in osteoclasts, we examined the intracellular expression of c-jun, ERK, Akt, MAPK p38, and NF-kB in BMM cells in the presence of RANKL and M-CSF (Control) and RANKL, M-CSF, and HxTME by western blot analysis. Time-course analysis (0–30 min) of western blots showed that the RANKL and M-CSF led to increased expression of c-Jun and MAPK p38 within 5–10 min post stimulation whereas Akt expression appeared unaltered in control BMM cells (Figures 3A,C). However, we observed that the treatment with HxTME significantly decreased the expression of c-Jun and Akt within 10 min of treatment. HxTME further retained its inhibitory effect on expression of c-Jun and Akt till 30 min of post stimulation when compared with RANKL, M-CSF, and HxTME treatment at 0 min and with only RANKL and M-CSF stimulated control (0 min). We also observed that the expression of MAPK p38 was decreased at 10 and 15 min after HxTME treatment and increased at 30 min when compared with HxTME treatment at 0 min and only RANKL and M-CSF stimulated BMM cells (0 min) (Figures 3A–D).

To further examine whether HxTME suppressed the phosphorylation of ERK, Akt and IKK β in osteoclasts, we investigated the expression of ERK, Akt and IKK β by western blot analysis using specific antibodies. Interestingly, HxTME suppressed phosphorylation of ERK, Akt, and IKK β at 30 min of HxTME treatment compared with RANKL and M-CSF stimulated control. In RANKL and M-CSF stimulated control, phosphorylation of ERK, IKK β, and Akt was detected unchanged at 0–30 min (Figure 3C). HxTME also prevented the degradation of IKK β in BMM cells stimulated with RANKL and M-CSF at 10 min compared with the RANKL and M-CSF stimulated control (Figure 3D). Altogether, our result suggests that HxTME inhibits the expression of the intermediates of RANK-RANKL signaling pathway.

### Effects of HxTME Fraction on Intracellular Calcium (${\text{Ca}}_{2}^{+}$)i in BMM Cells

**Figure 4:**
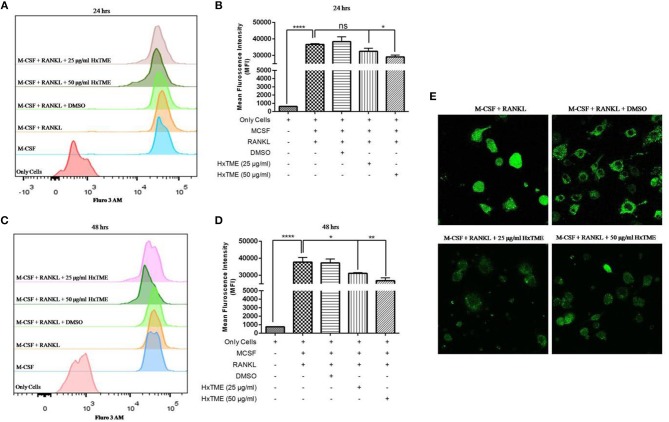
HxTME inhibits RANKL induced on intracellular calcium levels. Isolated BMM cells were cultured with M-CSF (30 ng/ml) and RANKL (30 ng/ml) along with indicated concentrations of HxTME for 24 and 48 h. Calcium [${\text{Ca}}_{2}^{+}$]i levels were measured using Fluo 3 AM dye on following day. Cells were acquired on FACS Aria. The data was analyzed using FlowJo software and represented as mean fluorescence intensity (MFI) after 24 h **(A,B)** and 48 h **(C,D)**. **(E)** Ca^2+^ imaging of RANKL and M-CSF stimulated BMM cells treated with HxTME for 48 h. All experiments were performed at least three times. All values were represented as Mean ± SEM. ^*^P ≤ 0.05; ^**^P ≤ 0.01 vs. M-CSF and RANKL treated control, and ^****^P ≤ 0.0001 vs. Only Cells.

RANKL induced NFATc1 activation increases intracellular calcium concentration which regulates osteoclast maturation and function (9). To examine the intracellular calcium levels, BMM cells were treated with HxTME in presence of RANKL for 24 and 48 h. We observed that, the MFI was decreased in RANKL stimulated BMM cells treated at concentration of 25 μg/ml (32552 MFI, non-significant) and 50 μg/ml (29108 MFI, *P* < 0.05) of HxTME as compared to only RANKL stimulated BMM cells (36681 MFI) after 24 h (Figures 4A,B). However, after 48 h of HxTME treatment MFI at concentration of 25 μg/ml (31130 MFI, *P* < 0.05) and 50 μg/ml (28839 MFI, *P* < 0.01) was significantly reduced compared to the control (Figures 4C,D). This observation was confirmed by confocal imaging for intracellular calcium using Fluo 3 AM staining of BMM cells treated with RANKL, M-CSF, and HxTME (Figure 4E). Our results revealed that HxTME significantly suppressed intracellular calcium concentration in BMM cells which plays a key role in supporting osteoclast differentiation.

### HxTME Prevents Bone Resorption in Ovariectomized Mice

**Figure 5:**
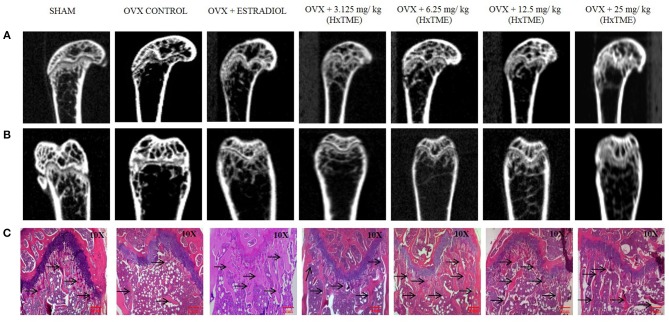
HxTME protects against ovariectomy induced bone loss by suppressing osteoclast activity. After ovariectomy and Sham operation mice were randomly divided into 7 indicted groups. OVX mice were treated with oral supplementation of HxTME for another 5 weeks. Later, all mice were euthanized for microCT imaging and bone histomorphometric analysis. **(A,B)** Representative coronal and saggital view of trabecular bone from the femur of Sham mice, OVX mice, and OVX treated with Estradiol, HxTME (3.125, 6.25, 12.5, and 25 mg/kg of body weight). **(C)** Representative images of decalcified bone stained with H&E from Sham mice, OVX mice, and OVX treated with Estradiol, HxTME (3.125, 6.25, 12.5, and 25 mg/kg of body weight). Experiments were repeated twice. Magnification at 10X and Arrow indicates trabecular processes, scale bar = 1mm.

The ovariectomized (OVX) mice mimic the changes in bone metabolism as observed in postmenopausal osteoporosis in humans. In our study, the female Swiss albino mice were bilaterally ovariectomized and orally fed with HxTME for a period of 5 weeks. After 5 weeks of treatment with HxTME, mice were sacrificed, and femur bones were analyzed for histomorphometric and microCT measurements. Histomorphometric analysis of femur bone of OVX group showed porous, sparse, disrupted trabecular bone with reduced trabecular number, and thickness as compared to Sham control group. Our data suggest that there is reduced endocortico-trabecular connectivity within the femur bone of OVX mice compared with Sham control. It was observed that there was restoration of the trabecular network and increased endocortico-trabecular connectivity after oral administration of HxTME in ovariectomized mice at all concentrations (Figure 5C). It was also observed that there was an increase in the trabeculation in the bone marrow space and reunion of the trabeculae in the HxTME treated group. Histological assessment confirmed that HxTME induced the formation of new cancellous bone in the femur comparable to that seen in the Sham control (Figure 5C).

Micro-CT images showed that the microstructure of the femur trabecular region in the OVX group was interrupted by the loss of trabecular interconnections with increased space compared with Sham control (Figures 5A,B). Detailed micro-CT analysis (Table 1) demonstrated that BMD was found to be decreased in OVX (373.12 mg/cc ± 7.24) group as compared with Sham control group (452.70 mg/cc ± 22.54). Estradiol (532.60 mg/cc ± 24.10, *P* < 0.0001) and HxTME at 3.125 mg/kg (452.95 mg/cc ± 9.57, *P* < 0.05) group showed significant escalation in BMD, whereas 6.25 mg/kg (396.69 mg/cc ± 24.40), 12.5 mg/kg (441.99 mg/cc ± 8.86), and 25 mg/kg (423.45 mg/cc ± 7.02) of HxTME also showed increase in BMD but was statistically non-significant as compared to Sham control group. Similarly, the Tb. Th of OVX group (0.017 mm ± 0.03) showed a marked decrease than Sham (0.93 mm ± 0.10). There was an apparent elevation in Tb. Th after the HxTME treatment at concentration of 25 mg/kg (0.96 mm ± 0.01, *P* < 0.01) and 3.125 mg/kg (0.98 mm ± 0.04) compared to the Sham control group. In addition, results also demonstrated that the trabecular space or separation was increased in the OVX (1.74 mm ± 0.03) group compared to Sham group (1.56 mm ± 0.01) suggesting that ovariectomy induced porosity and bone micro-architectural degradation. Tb. Sp was found to be significantly diminished in HxTME treated mice at concentrations of 12.5 mg/kg (1.25 mm ± 0.11, *P* < 0.05) and 6.25 mg/kg (1.30 mm ± 0.03, *P* < 0.05) as compared to the OVX. Simultaneously, connectivity density of OVX (0.0375 mm^−3^ ± 0.0025) group was reduced as compared to Sham control group (0.0454 mm^−3^ ± 0.002), whereas it was notably attenuated by Estradiol (0.0856 mm^−3^ ± 0.0029, *P* < 0.0001) treatment in ovariectomized mice. Bone volume density (BV/TV) did not show any major changes in HxTME treated and control groups. Specifically, micro-CT results demonstrated that ovariectomy induced changes in BMD, Tb.Th., and Tb.Sp. and connectivity density were significantly inhibited by the administration of HxTME.

**Table 1:** Effect of HxTME on bone micro-architecture analyzed by micro-CT.

**Groups**	**Bone mineral density (mg/cc)**	**BV/TV (mm^**3**^)**	**Tb.Th. (mm)**	**Tb.Sp. (mm)**	**Connectivity density (mm^**−3**^)**
Sham	452.70 ± 22.54	0.55 ± 0.03	0.93 ± 0.10	1.56 ± 0.01	0.0454 ± 0.002
OVX	373.12 ± 7.24	0.50 ± 0.01	0.017 ± 0.03	1.74 ± 0.03	0.0375 ± 0.0025
Estradiol	532.60 ± 24.10^***^	0.52 ± 0.01	0.85 ± 0.05	1.38 ± 0.18	0.0856 ± 0.0029^****^
25 mg/kg	423.45 ± 7.02	0.52 ± 0.003	0.96 ± 0.01^**^	1.76 ± 0.03	0.0354 ± 0.0001
12.5 mg/kg	441.99 ± 8.86	0.51 ± 0.02	0.76 ± 0.02	1.25 ± 0.11^*^	0.0537 ± 0.0061
6.25 mg/kg	396.69 ± 24.40	0.49 ± 0.03	0.79 ± 0.02	1.30 ± 0.03^*^	0.0577 ± 0.0002
3.125 mg/kg	452.95 ± 9.57^*^	0.50 ± 0.02	0.98 ± 0.04^**^	1.56 ± 0.09	0.0358 ± 0.0051

*Ovariectomized mice were treated with Estradiol and/or HxTME fraction for 5 weeks. Measurements of Bone mineral density (BMD), bone volume/tissue volume (BV/TV), trabecular thickness (Tb. Th), trabecular separation (Tb. Sp), and connectivity density were determined from micro-CT software. All values are expressed as mean ± SEM*.

* P ≤ 0.05,

** P ≤ 0.01,

*** P ≤ 0.001, and

**** *P ≤ 0.0001 vs. OVX*.

We examined the body weight of all groups of mice during the HxTME treatment (Supplementary Figure 1A). It was observed that there was no net change in body weight of ovariectomized mice. We also studied the uterine weight in treated groups, as the uterine tissue is a receptive site of estrogen. The uterine weight of the OVX group was found to be less than Sham control group (Supplementary Figures 1B,C). We observed that relative uterine weight at concentration of 12.5 mg/kg (0.152 gm, *p* < 0.01); 25 mg/kg (0.169 gm, *p* < 0.01) of HxTME treatment and estradiol control (0.146 gm, *p* < 0.01) had significantly increased as compared to the OVX group (0.0467 gm). This indicates that HxTME supplementation maintained uterine weight in ovariectomized mice which was decreased after ovariectomy. Histopathological observation of uteri of Sham group showed tortuous endometrium with columnar epithelium and large endometrial glands. In contrast, the OVX group showed a decrease in the number and size of uterine glands which appeared atrophied with closely packed stroma. The HxTME treated groups showed an increase in the number and size of the uterine glands as compared to the Sham group (Supplementary Figure 1C). Thus, collectively it proves that the oral supplementation of HxTME in ovariectomized mice had a beneficial effect on the uterus without inducing any harmful effects.

### Raman Spectroscopic Analysis of Bone Mineralization After HxTME Treatment

**Figure 6:**
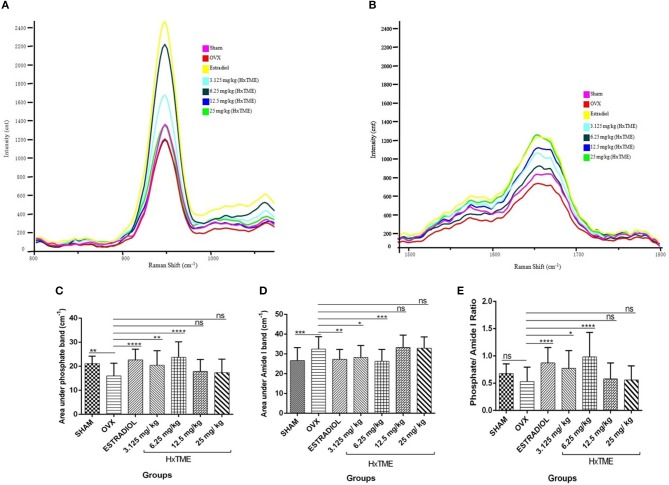
HxTME reversed ovariectomy induced mineral changes in bones. **(A)** Raman spectra of Phosphate (~959 cm^−1^) and **(B)** Amide I (1,665 cm^−1^) band of femur bones from control and HxTME treated groups of mice. Spectra were recorded in distal and shaft region at different points by using Raman microscope WITec alpha300RS (WITec GmbH, Ulm, Germany). Laser power at the specimen was 28 mW and spectra were integrated for 5 s and averaged over 10 accumulations. **(C)** Area under 960 cm^−1^ and **(D)** area under 1,665 cm^−1^ band against number of spectra was analyzed. Band fitting was performed on vector normalized and baseline corrected spectra using Gaussian function. Fitting was undertaken using band fitting algorithm of GRAMS/AI 7.02 (Thermo Electron Corporation) software. **(E)** Mineral to matrix ratio (Phosphate/Amide I) of Raman bands area under the curve was plotted from control and HxTME treated groups. All values were expressed in average ± SD. ^*^P ≤ 0.05, ^**^P ≤ 0.01, ^***^P ≤ 0.001, and ^****^P ≤ 0.0001 vs. OVX. ns; non-significant.

Several studies have shown that Raman spectroscopy can detect compositional changes in osteoporotic bones and also examines the relationship between the changes in osteoporotic bone composition with alternations in bone material properties (19, 20). In the present study, we have explored rapid and non-invasive Raman spectroscopic method to investigate the phosphate (~959 cm^−1^) and amide I band (1,665 cm^−1^) intensity in femur bones of all groups of control and HxTME treated mice. The results suggest that phosphate intensity was lowest in OVX group as compared to Sham group while it was enhanced in estradiol, 6.25 and 3.125 mg/kg of HxTME treated groups (Figure 6A). Amide I band intensity was also found to be lowest in the OVX group than Sham group. For the HxTME at a concentration of 25, 12.5, 6.25, 3.125 mg/kg and estradiol control groups the intensity of amide I (1,665 cm^−1^) peak was higher than that from the OVX group (Figure 6B).

We further calculated the area under the phosphate band to study the mineral crystallinity of bone. The average value of area under the phosphate band of OVX (15.97) was lower than that from estradiol control (22.70, *P* < 0.0001), 3.125 mg/kg (20.44, *P* < 0.01), and 6.25 mg/kg (23.70, *P* < 0.0001) of HxTME treated group. However, 12.5 mg/kg (17.90, ns) and 25 mg/kg (17.39, ns) concentration of HxTME showed a marginal increase in area under the curve of phosphate band (Figure 6C). Raman data demonstrated that the phosphate content was preserved in bones of OVX mice after oral administration of HxTME which was reduced in ovariectomized mice group. Simultaneously, the area under the amide I showed significant decrease in Estradiol control (27.35, *P* < 0.01), 3.125 mg/kg (28.28, *P* < 0.05), and 6.25 mg/kg (26.31, *P* < 0.001) of HxTME treated groups as compared with OVX group (Figure 6D).

We further studied the mineral to matrix content to compare the degree of inorganic and organic content between the normal and osteoporotic bone. The mineral to matrix ratio (phosphate/amide I) was significantly lower in the OVX group (0.5311) than Sham (0.6775) group. Results indicate that the phosphate /amide I ratio of estradiol (0.8730, *P* < 0.0001), 3.125 mg/kg (0.7734, *P* < 0.05), and 6.25 mg/kg (0.9831, *P* < 0.0001) of HxTME were significantly higher than the OVX group (Figure 6E). Raman analysis demonstrated that ovariectomy induced changes in mineral and organic content were significantly reversed by HxTME treatment.

### Effect of HxTME on Lymphocyte Proliferation Induced by T Cell Mitogens

**Figure 7:**
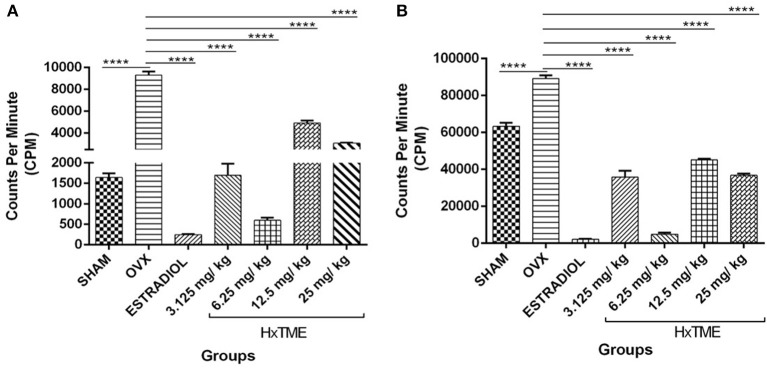
HxTME significantly reduced T cell proliferation. Ovariectomized mice were orally fed with HxTME for a period of 5 weeks. Mice were sacrificed after completion of oral supplementation and splenocytes were isolated from Sham, OVX, estradiol, and HxTME treated mice groups (3.125, 6.25, 12.5, and 25 mg/kg). Splenocytes isolated from these mice were stimulated with T cell mitogens **(A)** PMA/Io and **(B)** PHA for 24 h and the proliferation was determined by ^3^H-thymidine incorporation assay and expressed as counts per minute (CPM). All values were represented in Mean ± SD. ^****^P ≤ 0.0001 vs. OVX.

To evaluate the functionality of splenic lymphocytes from OVX and HxTME treated OVX mice, *in vitro* cell proliferation assay was carried out in response to stimulation with PMA/Io (Figure 7A) and PHA (Figure 7B). The proliferation of splenic lymphocytes in OVX group was highest in response to stimulation with PMA/Io and PHA as compared to Sham control. This increased proliferation in response to PMA/Io and PHA in OVX mice group was completely abolished by the treatment with estradiol (*p* < 0.0001) in ovariectomized mice. However, HxTME treatment at concentration of 3.125, 6.25, 12.5, and 25 mg/kg also showed a significant decrease in proliferative response to both PHA and PMA/Io as compared to lymphocytes of OVX mice. A marked decrease in lymphocyte proliferation in response to stimulation with PMA/Io and PHA was observed with 6.25 mg/kg of HxTME. Overall results demonstrated that the increased T cell proliferative response after ovariectomy was significantly attenuated by HxTME treatment.

### HxTME Attenuates Bone Turnover in Ovariectomized Mice

**Figure 8:**
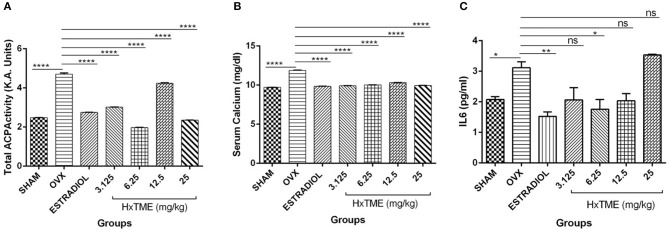
HxTME attenuates bone turnover markers in ovariectomized Mice. Blood was collected from control mice, OVX mice treated with HxTME, and estradiol after 5 weeks of oral administration and serum was separated. **(A)** The levels of serum ACP, **(B)** serum calcium, and **(C)** IL6 were measured. Cytokine levels of IL6 was analyzed and measured by Cytometric bead array. Data presented as a concentration in pg/ml. All experiments were repeated at least three times. All values are expressed as mean ± SD. ^*^P ≤ 0.05, ^**^P ≤ 0.01, and ^****^P ≤ 0.0001 vs. OVX.

Biochemical markers of bone turnover are products released by bone cells particularly osteoclasts and osteoblasts. At the end of the HxTME treatment to ovariectomized mice groups, serum levels of Acid phosphatase, calcium, and IL6 were examined. Total Acid phosphatase activity after HxTME treatment at concentration of 3.125 mg/kg (3.016 K.A. Units, *p* < 0.0001), 6.25 mg/kg (1.969 K.A. Units, *p* < 0.0001), 12.5 mg/kg (4.242 K.A. Units, *p* < 0.0001), 25 mg/kg (2.348 K.A. Units, *p* < 0.0001), and in Estradiol control (2.759 K.A. Units, *p* < 0.0001) was significantly decreased as compared to the OVX group (4.703 K.A. Units) (Figure 8A). It was observed that acid phosphatase activity, markedly increased in OVX group as compared to Sham group. However, we also found that the serum calcium levels in OVX group were increased after ovariectomy suggesting the increased amount of calcium in the serum of OVX mice was due to the active bone resorption. This increased calcium level was considerably restrained by oral administration of HxTME at concentration of 3.125 mg/kg (9.972 mg/dl, *p* < 0.0001), 6.25 mg/kg (10.03 mg/dl, *p* < 0.0001), 12.5 mg/kg (10.32 mg/dl, *p* < 0.0001), 25 mg/kg (9.947 mg/dl, *p* < 0.0001), and estradiol control (9.838 mg/dl, *p* < 0.0001) as compared to the OVX mice group (11.88 mg/dl) (Figure 8B). Overall, these results suggested that loss of calcium brings detrimental changes in the skeletal system which was markedly attenuated by HxTME supplementation in ovariectomized mice. Similarly, serum concentration of Acid phosphatase was suppressed by oral administration of HxTME providing evidence that HxTME treatment was able to reduce the bone turnover rate altered due to estrogen deficiency.

Cytokines are known to have a regulatory role in osteoclast formation and function. In post-menopausal women's, pro-osteoclastic cytokine such as IL6 is increased abundantly due to estrogen deficiency which encourages osteoclastogenesis and causes excessive osteolysis. In the present study, we measured the levels of IL6 in sera of Sham, OVX, Estradiol and HxTME treated mice groups. We observed that the serum level of IL6 was higher in OVX mice group compared to the Sham control group. This increased level of IL6 was suppressed by Estradiol (*p* < 0.01) and HxTME at concentration 3.125–12.5 mg/kg with the difference been statistically significant at 6.25 mg/kg (*p* < 0.05) in ovariectomized mice (Figure 8C). Collectively the data demonstrate that a pro-osteoclastic cytokine, IL6 was markedly reduced upon treatment with HxTME.

### Effect of HxTME on Immune Cells in Ovariectomized Mice

In order to understand the effect of HxTME on splenocyte subsets (splenic lymphocytes), spleens were isolated from all mice groups after 5 weeks of treatment and stained for cell surface markers such as CD3^+^, CD4^+^, CD8^+^, and CD19^+^ for multicolor flow cytometry. The data demonstrated that HxTME treatment did not change overall percentage population of T cell and B cell subset and are comparable to Sham control group (Supplementary Figures 2A,B).

**Figure 9:**
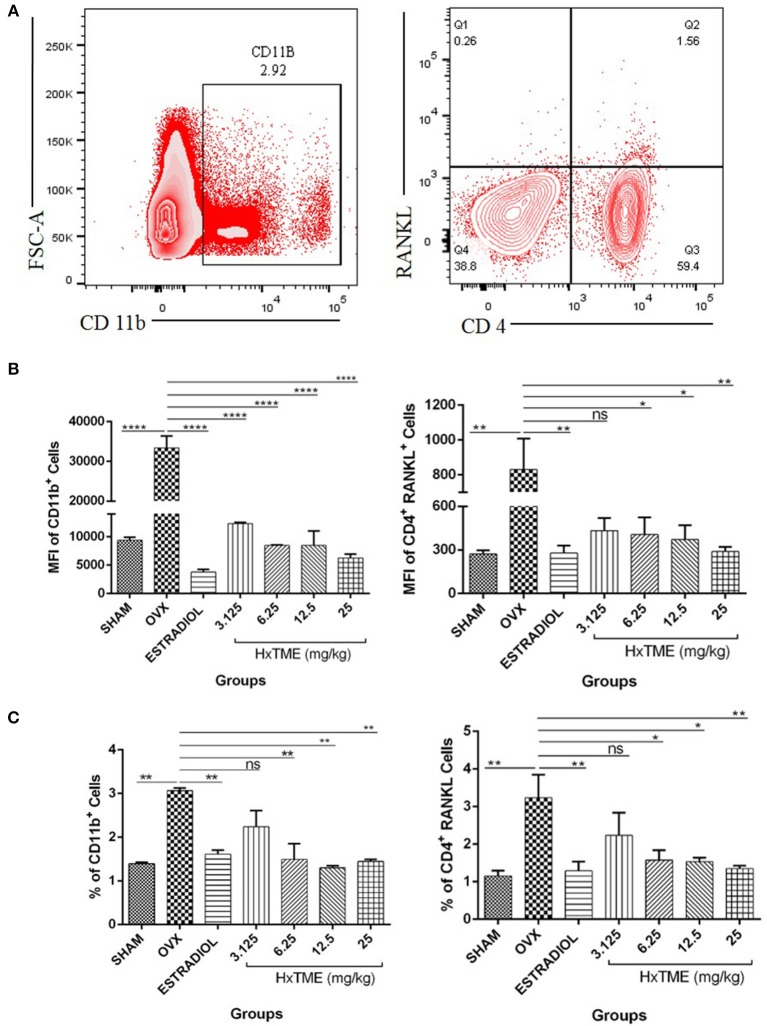
HxTME modulates immune response in ovariectomized mice. **(A)** Gating strategies to analyse the percentage and Mean fluorescence Intensity of CD11b^+^ and CD4^+^ RANKL T cells subsets in the splenocytes isolated from Sham, OVX, Estradiol, and HxTME treated OVX mice groups. **(B)** Bar plots show the Mean fluorescence Intensity CD11b^+^ and Mean fluorescence Intensity of CD4^+^ RANKL^+^ T cells. **(C)** Bar plots show the percentage of total CD11b^+^ and CD4^+^ RANKL T cells. All values are expressed as mean ± SEM. ^*^P ≤ 0.05, ^**^P ≤ 0.01, and ^****^P ≤ 0.0001 vs. OVX.

We further examined the presence of CD11b^+^ cells in splenocytes isolated from HxTME treated, OVX and Control mice groups by flow cytometry. Immunophenotyping data showed that the MFI of CD11b^+^ cells in OVX (33,392 ± 2,971) was increased compared to the Sham group (9,361 ± 549; *p* < 0.0001) (Figures 9A,B). There was a significant decrease in MFI of CD11b^+^ cells in HxTME treated ovariectomized mice at concentration of 3.125 mg/kg (12,312 ± 232; *p* < 0.0001), 6.25 mg/kg (8,469 ± 76; *p* < 0.0001), 12.5 mg/kg (8,477 ± 2,529; *p* < 0.0001), 25 mg/kg (6,280 ± 654; *p* < 0.0001), and Estradiol (3,761 ± 504; *p* < 0.0001) as compared to the OVX (33,392 ± 2,971) group (Figure 9B). In addition to this, the frequency of CD11b^+^ cells in OVX (3.06%) was increased compared to the Sham group (1.4%, *P* < 0.01) (Figure 9C). There was a significant decrease in percent population of CD11b^+^ cells in HxTME treated ovariectomized mice at concentration of 6.25mg/kg (1.49%; *p* < 0.01), 12.5 mg/kg (1.3%, *p* < 0.01), 25 mg/kg (1.4%; *p* < 0.01), and Estradiol (1.6%; *p* < 0.01) as compared to the OVX (3.06%) group (Figure 9C).

In parallel, we also studied the expression of another macrophage marker F4/80 in splenocytes isolated from Control, OVX, and HxTME treated ovariectomized mice groups by flow cytometry (Supplementary Figures 3A,B). It was observed that the F4/80 expression followed the same trend as CD11b. The increased expression of F4/80 in OVX mice (15744 MFI and 86.5%) was significantly reduced upon Estradiol (12854, MFI and 70.7%) and HxTME treated OVX mice at concentration of 3.125 mg/kg (13135, MFI and 77.4%), 6.25 mg/kg (12044, MFI and 75.5%), 12.5 mg/kg (11151, MFI and 73.3%), and 25 mg/kg (10327 MFI and 69.7%). We also observed co-expression of CD11b and F4/80 in splenocytes of HxTME treated, OVX and Control Mice. There was a significant decrease in percentage population of CD11b^+^ F4/80^+^ in HxTME treated mice groups at concentration of 3.125 mg/kg (1.2%, *P* < 0.05), 6.25 mg/kg (0.8%, *P* < 0.05), 12.5 mg/kg (1.66%), and 25 mg/kg (1.5%) compared to OVX mice (4.76%).

We have also examined the surface expression of RANKL on CD4^+^ T cells in splenocytes. We detected that OVX (830 ± 177, MFI and 3.2%) group showed higher expression of RANKL on CD4^+^ T cells as compared to Sham control (271.7 ± 25, MFI and 1.15%) (Figures 9B,C). HxTME treatment significantly reduced the MFI and percentage levels of RANKL expressing CD4^+^ T cells in 6.25, 12.5, and 25 mg/kg concentrations by 406.3 ± 118.6 (1.58%), 371.8 ± 98 (1.53%), and 289.7 ± 32.27 (1.35%), respectively (Figures 9B,C), except 3.125 mg/kg (432.7 ± 87; 2.24%) of HxTME. Estradiol also showed significant decrease in RANKL expression i.e., 278.3 ± 50.89 (1.29%) as compared to the OVX group. Results indicate that HxTME supplementation in ovariectomized mice down-regulates the RANKL expression on T cells.

### Fatty Acid Profiling of HxTME Fraction Through GCMS Analysis

GCMS analysis of HxTME revealed that the main fatty acid was Palmitic acid (39.5%), followed by Octadecenoic acid (10.19%), Eicosapentaenoic acid (EPA) (12.27%), Eicosatetraenoic acid (ETA) (8.7%), terpenes (1.67%), and Adrenic acid (4.9%) as summarized in Table 2. The fatty acid profile of HxTME showed a high percentage of saturated fatty acids (47.44%) due to the content of Palmitic acid (C16:0), which, alone, contributed with 39.5% of the total. Octadecenoic acid (C18:1n-3) was the major constituent among the monounsaturated fatty acids and Eicosapentaenoic (EPA; C20:5) and Eicosatetraenoic acid (ETA; 20:4) are among the polyunsaturated fatty acids (PUFAs). We have also calculated the ratio of omega (ω) 6/omega (ω) 3 in a fraction as it is important for metabolic and physiological health in humans. We found that the ratio of omega (ω) 6/omega (ω) 3 in HxTME was 1:2.5. Overall, GCMS study demonstrated that HxTME has a higher content of fatty acids including PUFAs with healthy omega 6/omega 3 ratios.

**Table 2:** GCMS analysis of HxTME.

**Fatty acids profile of HxTME**	**Lipid number**	**% peak area**
**Saturated (SFA)**		
Myristic acid, methyl ester	C14:0	1.68
Tridecanoic acid	C13:0	1.03
Pentadecylic acid	C15:0	1.18
Palmitic acid	C16:0	39.5
Margaric acid	C17:0	2.17
Stearic acid	C18:0	1.88
Total		**47.44**
**Polyunsaturated (PUFA)**		
Linoleic acid	C18:2	2.27
α-Linolenic acid	C18:3	1.4
Eicosatetraenoic acid (ETA)	C20:4	8.7
Eicosapentaenoic acid (EPA)	C20:5	12.27
Adrenic acid	C22:4	4.9
Linoleic acid	C18:2	1.69
Total		**31.23**
**Monounsaturated fatty acids**		
Octadecenoic acid	C18:1 n-3	10.19
Oleic acid	C18:1 cis-9	2.81
Total		**13.00**
**Terpenes**		1.67
Total		**1.67**
**Miscellaneous**		**6.67**

*Bold values indicate the percent peak area*.

## Discussion

Perturbations in bone remodeling are mostly caused by over-activation of osteoclasts leading to excessive bone destruction (21, 22). In adverse stages of postmenopausal osteoporosis, current treatment involves extensive use of anti-resorptive and anabolic agents (23). Administration of these agents reduces bone resorption by inhibiting the activity of osteoclasts through blocking RANK-RANKL interaction and enhancing new bone formation through stimulating osteoblast functioning (24). Although, most of the anti-resorptive agents have been developed to limit bone resorption in postmenopausal osteoporosis, their costs are not only too high to benefit a large population in the world but are also associated with adverse health effects. Consequently, it is necessary to develop novel natural entities with fewer side effects that can substitute for drugs used currently. The marine environment is a potential source of novel natural products with promising biological activities but is less explored for the treatment of bone-related diseases.

In the present study, we have investigated the anti-osteoclastogenic effects of fraction derived from crude TME and its molecular mechanism in RANK-RANKL signaling pathway. *In vitro* osteoclastogenesis and resorption pit analysis provides strong evidence that HxTME exerts a direct and dose-dependent effect on RANKL-induced osteoclast differentiation and bone resorption. Our study demonstrated that the HxTME impaired osteoclast formation and maturation process with a significant decrease in osteoclast resorption through disturbing actin ring formation. MMPs, Cathepsin k, and TRAP were actively expressed by mature osteoclasts (25). Interestingly, we found that HxTME showed a marked reduction in MMP2 and MMP9 expression suggesting that HxTME has not only an inhibitory role on MMP2 and MMP9 production but also suppressed osteoclasts activity.

RANK-RANKL interaction results in differentiation, fusion, activation, and survival of osteoclasts. This interaction recruits TRAF6, which is involved in activation of downstream signaling pathways such as ERK, JNK, NFκB, and p38 MAPKs pathways (26–28). In addition to this, RANKL also activates several transcription factors like c-Fos, NFATc1, and NFkB which are crucial for osteoclast differentiation (9). Particularly, NFATc1 is a master regulator of osteoclast differentiation which regulates a number of osteoclast-specific genes includes Cathepsin k, TRAP, Osteoclast-associated receptor (OSCAR), Calcitonin receptor, and c-FOS (29). NFATc1-deficient mice develop osteopetrosis due to impaired osteoclastogenesis but its ectopic expression in osteoclast precursor cells induces osteoclasts differentiation without RANKL (30). However, c-Fos, a member of the activator protein (AP-1) family of transcription factor plays an essential role in up-regulation of RANK expression in osteoclast precursors within the bone environment (31). Our data demonstrated that the HxTME treatment significantly down-regulates the expression of master transcriptional factors such as NFATc1 and c-FOS in RANKL-induced osteoclastogenesis and inhibited the expression of osteoclasts related genes; TRAP and Cathepsin k since they are downstream transcriptional targets of NFATc1.

In addition to RANKL, M-CSF plays a critical role in osteoclast differentiation which was well-demonstrated in mice with severe osteopetrosis due to lack of CSF-1 gene (*Csf* 1°^p^/*Csf* 1°^p^) (32). Mice were rendered osteopetrotic by administration of a neutralizing anti-CSF-1 antibody which displayed high bone mass phenotype, due to decreased growth rate and significant reduction in osteoclasts number (33). However, transgenic expression of cell-surface M-CSF (csCSF-1) and circulating CSF-1 corrected the gross defects in CSF-1-deficient osteopetrotic (*Csf* 1°^p^/*Csf* 1°^p^) mice (34). Recently, Hodge et al. showed that the M-CSF augments RANKL-induced activation of c-Fos and ERK 1/2 phosphorylation, but did not affect the activation of NFkB and NFATc1 and also revealed its unidentified role as a potent stimulator of mature osteoclasts resorbing activity mediated through ERK upstream of c-Fos (35). Therefore, in the present study, we cannot rule out that HxTME may also mediate its effect through M-CSF which induces osteoclast differentiation and cell signaling. The key events in RANKL signaling involves activation of MAPKs (ERK, p38, JNK), NFκB, and Akt kinases (36). Interestingly, we observed that the HxTME inhibits c-jun, Akt, MAPKp38, and ERK expression, and therefore has the potential to modulate both M-CSF and RANKL signaling in osteoclasts. Further studies using serum starvation and blocking of M-CSF would clearly establish the role of HxTME in modulation of M-CSF and RANKL signaling. It also inhibits phosphorylation and degradation of IKK β. Activated NFATc1 in presence of RANKL increases intracellular calcium concentration which regulates osteoclast maturation and function (37). Intriguingly, HxTME significantly suppressed intracellular calcium levels. This implies that HxTME altered the intracellular calcium levels which are subsequently involved in downregulation of osteoclasts-specific transcription factors, c-FOS and NFATc.

We further examined the therapeutic importance of HxTME *in vivo* in the ovariectomized mice model. Ovariectomy ceases the endogenous estrogen production which manifested into marked uterine atrophy. In the present study, the oral supplementation of HxTME did not show any adverse estrogenic effect. However, the uterine weight was maintained by HxTME supplementation suggesting an uterotrophic activity of HxTME. Till date, there are very few reports on marine extracts that have exhibited uterotrophic effect by maintaining the uterine weight which was reduced after ovariectomy. Recently, Li et al. demonstrated that the Du–Zhong–Wan water (DZW) extract of Chinese herb showed a beneficial effect on uterine weight and simultaneously preserved bone quality and strength and thereby exhibiting its anti-osteoporotic effect in ovariectomized rats (38).

Bone turnover markers are important tools to predict the dynamics of bone turnover in bone related metabolic disorders (39). Oral administration of HxTME attenuated the calcium loss in serum after ovariectomy indicating its physiological role in maintaining calcium levels. Serum concentration of Acid phosphatase was significantly high in the ovariectomized control mice suggesting a high bone turnover rate in OVX, which was markedly reduced after oral administration of HxTME. Ovariectomy has shown high remodeling by an increase in bone turnover markers which was prominently reduced by HxTME supplementation.

Studies have shown that the withdrawal of estrogen following ovariectomy induce perforation in trabecular plates and loss of connectivity density which results in the deterioration of trabecular bone micro-architecture (40–42). In the present study, oral administration of HxTME over a period of 5 weeks preserves bone micro-architecture and reduced bone deterioration through an increase in trabecular formation and reunion of trabeculae in ovariectomized mice. It was found that there was escalation in BMD, Tb. Th, and connectivity density with a decrease in Tb. Sp after HxTME treatment. Thus, HxTME preserves bone micro-architecture by maintaining bone volume density, connectivity index, and trabecular networking in ovariectomized mice. Raman spectroscopic study of bone showed decrease in the level of phosphate (959 cm^−1^) in bones of OVX mice. The phosphate band (~959 cm^−1^) represents the phosphate content which is not influenced by environmental factors. The width of the phosphate band (960 cm^−1^) is considered as a measure of mineral crystallinity of bone (43). The width of the phosphate band was found to be high in HxTME treated groups as compared to the ovariectomized mice group. However, analysis on collagen marker i.e., amide I band revealed that the area under the amide I of OVX group was higher than Sham. This increased collagen marker in ovariectomized mice was significantly reduced by HxTME treatment. Studies on cortical bone tissue of normal and ovariectomized mice proved that the Raman spectra in the amide I region was significantly increased in OVX treated samples as compared to control due to increase in elastic stress intensification in the mineral phase of OVX, thus proving degradation in the (elastic) energy-dissipative capacity of a diseased bone matrix (44). Decreased mineral/matrix (phosphate/amide I) ratio in OVX group suggested that the ovariectomy caused specific chemical changes in the bone sites, which indicates some degree of remodeling, especially in the trabecular bone sites (45). Mineral to matrix ratio was significantly increased in HxTME treated group than OVX group. The data suggested that ovariectomy-induced changes in mineral and organic content were significantly reversed by HxTME treatment.

The key molecules RANK and RANKL are expressed on activated T cells, dendrite cells, osteoclasts precursors, osteoblasts etc., and involved in cell survival and modulation of the immune system (46). Estrogen has been shown to affect T cells functions through an estrogen-mediated pathway and also modulate all T cell subsets that include CD4^+^ (Th1, Th2, Th17, and Tregs) and CD8^+^ T cells (47). Activated CD4^+^ T cells play a major role in osteoclast activation, mainly due to the expression of RANKL (48). Our data confirmed that the oral supplementation of the HxTME fraction to the ovariectomized mice downregulates RANKL expression on the CD4^+^T cells. Reports also suggested that increase expression of CD11b promote osteoclasts maturation through spleen tyrosine kinase (Syk) signaling pathway (49). It is also demonstrated that TNFα is responsible for induction of CD11b expressions of osteoclasts precursor cells through various signaling pathways (50). In this study, the percent population and MFI of CD11b^+^ cells were decreased in splenocytes of HxTME treated ovariectomized mice groups suggesting the possible therapeutic role of HxTME in osteolytic bone diseases.

The F4/80 is a specific marker for monocyte and macrophage. It is reported by Takeshita et al. that the F4/80 is also an important marker for osteoclast formation and osteoclasts were induced more from CD11b^−^ F4/80 dull fraction (51). However, the study conducted on dietary fish oil had shown that the proportion of red pulp macrophages, expressing F4/80, was lower in spleens from mice fed with the fish oil diet than in spleens from mice fed the corn oil diet (52). The present study also observed that the HxTME treatment to ovariectomized mice reduces the total expression of F4/80 and showed decreased expression of CD11b^+^ F4/80^+^ upon treatment of HxTME fraction to OVX mice.

It has been established that T cells and their products are main regulators for bone remodeling. Studies on animal models demonstrated that T cell deficient ovariectomized mice failed to induce bone loss and did not stimulate osteoclastogenesis which was reversed by reconstitution of T cells from wild type (WT) mice (53, 54). Activated T cells produce various cytokines such as IL6, IL1, IL17, TNFα, and RANKL which are majorly involved in osteoclastogenesis and bone loss. Lymphocytes also secrete certain inhibitory molecules during inflammations like OPG, IL4 and IFN-γ that directly prevents osteoclast formation (55). In the present study, we observed a marked decrease in IL6, pro-osteoclastic cytokine in HxTME treated ovariectomized mice. We also studied the serum levels of IFN γ in HxTME treated OVX mice, but no significant changes were observed in treated and control mice groups (data not shown). Thus, HxTME supplementation limits bone resorption via modulating the activation and RANKL expression of T cells and cytokine IL6 production. Ovariectomy-induced estrogen deficiency provokes T cell activation and promotes T cell proliferation, expansion, and their functions (56). HxTME significantly decreased the lymphocyte proliferative response to PMA/Io and PHA in ovariectomized mice. These results indicate that a marked increase in T cell proliferation after estrogen deficiency was significantly attenuated by HxTME.

HxTME fraction showed the presence of n-3 PUFAs, particularly Eicosatetraenoic acid (ETA) and Eicosapentaenoic acid (EPA) in relatively high amount which is considered to be essential functional food or nutraceutical for human health (57, 58). EPA and DHA are associated with optimizing osteoblastogenesis and inhibiting osteoclastogenesis by affecting the levels of pro-osteoclastic cytokines such as IL6, and TNFα (59, 60). Recently, the study conducted in the 1865 Spanish women, the dietary intake of long-chain omega3-PUFAs are positively associated with BMD at both the hips and the lumbar spine in normal and osteopenic women (61). These evidences highlight the importance of polyunsaturated fatty acid in the treatment of postmenopausal osteoporosis. We believe that ETA and EPA along with terpenes may be synergistically involved in possible anti-inflammatory, anti-osteoporotic activity, and in the mechanism of reversal of bone loss. Interestingly, HxTME showed the presence of rarely found ETA (n-3 PUFA) which may be linked with therapeutic potential against bone-related disease in humans. Although it was well-understood that the chemical constituents present in HxTME collectively elicited reversal of bone resorption, the action potential of individual compounds or molecules in it is unclear and needs further investigation.

In conclusion, HxTME directly inhibits osteoclast differentiation and maturation. It also suppresses pro-osteoclastic stimulus provided by T cells, thereby directly or indirectly inhibiting bone resorption. Furthermore, *in vivo* physio-chemical studies confirmed the beneficial effect of HxTME on skeletal health by preserving bone micro-architecture and mineral crystallinity in bilaterally ovariectomized mice model. Our preliminary data indicates that the HxTME treatment also increased osteoblasts differentiation and mineralization (data not shown). Marine nutraceuticals provide a myriad of bioactive molecules. These bioactive molecules have been used to prevent and cure various diseases such as obesity, cardiovascular diseases, arthritis etc for centuries. This study highlights the potential of HxTME to be used as a Marine nutraceutical and that may aid in the treatment of bone-related diseases.

## Ethics Statement

This study was carried out in accordance with the recommendations of Committee for the Purpose of Control and Supervision of Experiments on Animals (CPCSEA), Institutional Animal Ethics Committee (IAEC), ACTREC. The protocol was approved by the Institutional Animal Ethics Committee (IAEC), ACTREC.

## Author Contributions

SChi and MI conceived the project and designed the experiments. SCha performed the experiments and statistical analysis. SCha and SD conducted the animal study. SK conducted the osteoblasts culture and flow experiments. CK and PC acquired analyzed and interpreted Raman spectroscopy and microCT data. SChi, SCha, and MI analyzed and interpreted the data and drafted the manuscript. All authors have approved the final version of the manuscript.

### Conflict of Interest Statement

The authors declare that the research was conducted in the absence of any commercial or financial relationships that could be construed as a potential conflict of interest.

## Supplementary Material

Supplementary Figure 1 Effect of HxTME fraction on body weight, uterine weight, and histology of ovariectomized mice upon HxTME treatment. After 5 weeks of oral administration of HxTME fraction, **(A)** body weight was measured in control, estradiol, and treated groups of ovariectomized mice. Similarly, **(B)** uterine weight was also measured after euthanization of control, estradiol, and treated groups of ovariectomized mice. All values are expressed as mean ± SEM, ^**^*P* ≤ 0.01, ^***^*P* ≤ 0.001, and ^****^*P* ≤ 0.0001 vs. OVX. **(C)** Uterus was dissected out at the end of HxTME treatment. Uterus tissue was fixed in formalin, dehydrated in alcohol and paraffin sections were made. Sections were stained by using Haematoxylin and Eosin. Representative photomicrographs of uterine tissue were taken at 10X magnification. Scale bar = 1mm “E” (arrow) indicates uterine epithelium and “U” (circled) indicates uterine glands.

Supplementary Figure 2 Effect of HxTME on percentage population of T and B cell subsets in ovariectomized mice. **(A)** Gating strategies to analyse the percentage of T and B cell subsets in the splenocytes isolated from Sham, OVX, Estradiol, and HxTME treated OVX mice groups. **(B)** Bar plots show the percentage of total CD3^+^ T cells, CD3^+^CD4^+^ T cells, CD3^+^CD8^+^ T cells, and CD19^+^ cells. All values are expressed as mean.

Supplementary Figure 3 Effect of HxTME on F4/80 osteoclast precursor marker. **(A)** Gating strategies and graphical representation (Bar Plots) of percentage and Mean Fluorescence Intensity of F4/80^+^ cells in the splenocytes isolated from Sham, OVX, Estradiol, and HxTME treated OVX mice groups. **(B)** Gating strategies and graphical representation (Bar Plots) of percentage and Mean Fluorescence Intensity of CD11b^+^ F4/80^+^ cells in the splenocytes isolated from Sham, OVX, Estradiol, and HxTME treated OVX mice groups. All values are expressed as mean ± SEM. ^*^*P* ≤ 0.05, ^**^*P* ≤ 0.01, ^***^*P* ≤ 0.001, and ^****^*P* ≤ 0.0001 vs. OVX.

## References

[1] Liu H Luo T Tan J Li M Guo J. 'Osteoimmunology' offers new perspectives for the treatment of pathological bone loss. Curr Pharm Des. (2017) 23:6272–8. 10.2174/1381612823666170511124459 28494718

[2] Gambacciani M Levancini M. Management of postmenopausal osteoporosis and the prevention of fractures. Panminerva Med. (2014) 56:115–31. 24942322

[3] D'Amelio P Sassi F. Osteoimmunology: from mice to humans. Bonekey Rep. (2016) 5:802. 10.1038/bonekey.2016.29 27195109 PMC4870940

[4] Pisani P Renna MD Conversano F Casciaro E Di Paola M Quarta E. Major osteoporotic fragility fractures: risk factor updates and societal impact. World J Orthoped. (2016) 7:171–81. 10.5312/wjo.v7.i3.171 27004165 PMC4794536

[5] Weitzmann MN. Bone and the immune system. Toxicol Pathol. (2017) 45:911–24. 10.1177/0192623317735316 29046115 PMC5749254

[6] Balakrishnan B Indap MM Singh SP Krishna CM Chiplunkar SV. Turbo methanol extract inhibits bone resorption through regulation of T cell function. Bone. (2014) 58:114–25. 10.1016/j.bone.2013.10.008 24140785

[7] Guerrini MM Takayanagi H. The immune system, bone and RANKL. Arch Biochem Biophys. (2014) 561:118–23. 10.1016/j.abb.2014.06.003 24929185

[8] Park JH Lee NK Lee SY. Current understanding of RANK signaling in osteoclast differentiation and maturation. Mol Cells. (2017) 40:706–713. 10.14348/molcells.2017.0225 29047262 PMC5682248

[9] Kim JH Kim N. Regulation of NFATc1 in osteoclast differentiation. J Bone Metab. (2014) 21:233–41. 10.11005/jbm.2014.21.4.233 25489571 PMC4255043

[10] Boyce BF Yamashita T Yao Z Zhang Q Li F Xing L. Roles for NF-kappaB and c-Fos in osteoclasts. J Bone Miner Metab. (2005) 23 (Suppl):11–5. 10.1007/BF03026317 15984408

[11] Chaugule SR Indap MM Chiplunkar SV Marine natural products: new avenue in treatment of osteoporosis. Front Mar Sci. (2017) 4:384 10.3389/fmars.2017.00384

[12] Blunt JW Carroll AR Copp BR Davis RA Keyzers RA Prinsep MR. Marine natural products. Nat Prod Rep. (2018) 35:8–53. 10.1039/C7NP00052A 29335692

[13] Shin HJ Choi BK Trinh PTH Lee HS Kang JS Van TTT. Suppression of RANKL-induced osteoclastogenesis by the metabolites from the marine fungus *Aspergillus flocculosus* isolated from a sponge *Stylissa* sp. Mar Drugs. (2018) 16:E14. 10.3390/md16010014 29304006 PMC5793062

[14] Bucar F Wube A Schmid M. Natural product isolation – how to get from biological material to pure compounds. Nat Prod Rep. (2013) 30:525–45. 10.1039/c3np20106f 23396532

[15] Kim J-Y Park SH Oh HM Kwak SC Baek JM Lee MS. *Ampelopsis brevipedunculata* extract prevents bone loss by inhibiting osteoclastogenesis *in vitro* and *in vivo*. Molecules. (2014) 19:18465–78. 10.3390/molecules191118465 25397737 PMC6270923

[16] Bradley EW Oursler MJ. Osteoclast culture and resorption assays. In: Westendorf JJ editor. Osteoporosis: Methods and Protocols. Totowa, NJ: Humana Press (2008). p. 19–35. 10.1007/978-1-59745-104-8_2 18463808

[17] Kim J-Y Cheon YH Oh HM Rho MC Erkhembaatar M Kim MS Oleanolic acid acetate inhibits osteoclast differentiation by downregulating PLCγ2–Ca2+-NFATc1 signaling, and suppresses bone loss in mice. Bone. (2014) 60:104–11. 10.1016/j.bone.2013.12.013 24361669

[18] da Paz LH de Falco V Teng NC dos Reis LM Pereira RM Jorgetti V. Effect of 17beta-estradiol or alendronate on the bone densitometry, bone histomorphometry and bone metabolism of ovariectomized rats. Braz J Med Biol Res. (2001) 34:1015–22. 10.1590/S0100-879X2001000800007 11471040

[19] Boskey AL Donnelly E Boskey E Spevak L Ma Y Zhang W. Examining the relationships between bone tissue composition, compositional heterogeneity and fragility fracture: a matched case controlled FTIRI study. J Bone Miner Res. (2016) 31:1070–81. 10.1002/jbmr.2759 26636271 PMC4862946

[20] Mandair GS Morris MD. Contributions of Raman spectroscopy to the understanding of bone strength. Bonekey Rep. (2015) 4:620. 10.1038/bonekey.2014.115 25628882 PMC4296861

[21] Roux C Briot K. Osteoporosis in 2017: addressing the crisis in the treatment of osteoporosis. Nat Rev Rheumatol. (2018) 14:67–8. 10.1038/nrrheum.2017.218 29323345

[22] Novack DV Mbalaviele G. Osteoclasts, key players in skeletal health and disease. Microbiol Spectr. (2016) 4:1–19. 10.1128/microbiolspec.MCHD-0011-2015 27337470 PMC4920143

[23] Tabatabaei-Malazy O Salari P Khashayar P Larijani B. New horizons in treatment of osteoporosis. DARU J Pharm Sci. (2017) 25:2. 10.1186/s40199-017-0167-z 28173850 PMC5297185

[24] Lovato C Lewiecki EM. Emerging anabolic agents in the treatment of osteoporosis. Expert Opin Emerg Drugs. (2017) 22:247–57. 10.1080/14728214.2017.1362389 28756709

[25] Delaissé JM Andersen TL Engsig MT Henriksen K Troen T Blavier L. Matrix metalloproteinases (MMP) and cathepsin K contribute differently to osteoclastic activities. Microsc Res Tech. (2003) 61:504–13. 10.1002/jemt.10374 12879418

[26] Jules J Ashley JW Feng X. Selective targeting of RANK signaling pathways as new therapeutic strategies for osteoporosis. Expert Opin Ther Targets. (2010) 14:923–34. 10.1517/14728222.2010.511179 20678025 PMC2929902

[27] Kim JH Kim N. Signaling pathways in osteoclast differentiation. Chonnam Med J. (2016) 52:12–7. 10.4068/cmj.2016.52.1.12 26865996 PMC4742606

[28] Feng X. RANKing intracellular signaling in osteoclasts. IUBMB Life. (2005) 57:389–95. 10.1080/15216540500137669 16012047

[29] Song I Kim JH Kim K Jin HM Youn BU Kim N. Regulatory mechanism of NFATc1 in RANKL-induced osteoclast activation. FEBS Lett. (2009) 583:2435–40. 10.1016/j.febslet.2009.06.047 19576893

[30] Takayanagi H Kim S Koga T Nishina H Isshiki M Yoshida H. Induction and activation of the transcription factor NFATc1 (NFAT2) integrate RANKL signaling in terminal differentiation of osteoclasts. Dev Cell. (2002) 3:889–901. 10.1016/S1534-5807(02)00369-6 12479813

[31] Arai A Mizoguchi T Harada S Kobayashi Y Nakamichi Y Yasuda H. Fos plays an essential role in the upregulation of RANK expression in osteoclast precursors within the bone microenvironment. J Cell Sci. (2012) 125(Pt 12):2910–7. 10.1242/jcs.099986 22454522

[32] Dai XM Zong XH Sylvestre V Stanley ER. Incomplete restoration of colony-stimulating factor 1 (CSF-1) function in CSF-1-deficient Csf1op/Csf1op mice by transgenic expression of cell surface CSF-1. Blood. (2004) 103:1114–23. 10.1182/blood-2003-08-2739 14525772

[33] Wei S Lightwood D Ladyman H Cross S Neale H Griffiths M. Modulation of CSF-1-regulated post-natal development with anti-CSF-1 antibody. Immunobiology. (2005) 210:109–19. 10.1016/j.imbio.2005.05.005 16164017

[34] Ryan GR Dai XM Dominguez MG Tong W Chuan F Chisholm O. Rescue of the colony-stimulating factor 1 (CSF-1)-nullizygous mouse (Csf1(op)/Csf1(op)) phenotype with a CSF-1 transgene and identification of sites of local CSF-1 synthesis. Blood. (2001) 98:74–84. 10.1182/blood.V98.1.74 11418465

[35] Hodge JM Collier FM Pavlos NJ Kirkland MA Nicholson GC. M-CSF potently augments RANKL-induced resorption activation in mature human osteoclasts. PLoS ONE. (2011) 6:e21462. 10.1371/journal.pone.0021462 21738673 PMC3126821

[36] Lee K Chung YH Ahn H Kim H Rho J Jeong D. Selective regulation of MAPK signaling mediates RANKL-dependent osteoclast differentiation. Int J Biol Sci. (2016) 12:235–45. 10.7150/ijbs.13814 26884720 PMC4737679

[37] Kim H Kim T Jeong BC Cho IT Han D Takegahara N. Tmem64 modulates calcium signaling during RANKL-mediated osteoclast differentiation. Cell Metab. (2013) 17:249–60. 10.1016/j.cmet.2013.01.002 23395171 PMC3569742

[38] Li F Yang X Bi J Yang Z Zhang C. Antiosteoporotic activity of Du-Zhong-Wan water extract in ovariectomized rats. Pharm Biol. (2016) 54:1857–64. 10.3109/13880209.2015.1133657 26760929

[39] Shetty S Kapoor N Bondu JD Thomas N Paul TV. Bone turnover markers: Emerging tool in the management of osteoporosis. Indian J Endocrinol Metab. (2016) 20:846–52. 10.4103/2230-8210.192914 27867890 PMC5105571

[40] Kinney JH Haupt DL Balooch M Ladd AJ Ryaby JT Lane NE. Three-dimensional morphometry of the L6 vertebra in the ovariectomized rat model of osteoporosis: biomechanical implications. J Bone Miner Res. (2000) 15:1981–91. 10.1359/jbmr.2000.15.10.1981 11028451

[41] Kim CH Takai E Zhou H von Stechow D Müller R Dempster DW. Trabecular bone response to mechanical and parathyroid hormone stimulation: the role of mechanical microenvironment. J Bone Miner Res. (2003) 18:2116–25. 10.1359/jbmr.2003.18.12.2116 14672346

[42] Aslam MN Kreider JM Paruchuri T Bhagavathula N DaSilva M Zernicke RF. A mineral-rich extract from the red marine algae *Lithothamnion calcareum* preserves bone structure and function in female mice on a Western-style diet. Calcif Tissue Int. (2010) 86:313–24. 10.1007/s00223-010-9340-9 20180099 PMC2877502

[43] Morris MD Mandair GS. Raman assessment of bone quality. Clin Orthop Relat Res. (2011) 469:2160–9. 10.1007/s11999-010-1692-y 21116756 PMC3126952

[44] Pezzotti G Rondinella A Marin E Zhu W Aldini NN Ulian G. Raman spectroscopic investigation on the molecular structure of apatite and collagen in osteoporotic cortical bone. J Mech Behav Biomed Mater. (2017) 65:264–73. 10.1016/j.jmbbm.2016.08.030 27608424

[45] de Souza RA Jerônimo DP Gouvea HA Oliveira MX Souza MT Miranda H Fourier-transform Raman spectroscopy study of the ovariectomized rat model of osteoporosis. Open Bone J. (2010) 2:24–31. 10.2174/1876525401002010024

[46] Walsh MC Choi Y. Biology of the RANKL–RANK–OPG system in immunity, bone, and beyond. Front Immunol. (2014) 5:511. 10.3389/fimmu.2014.00511 25368616 PMC4202272

[47] Khan D Ansar Ahmed S. The immune system is a natural target for estrogen action: opposing effects of estrogen in two prototypical autoimmune diseases. Front Immunol. (2015) 6:635. 10.3389/fimmu.2015.00635 26779182 PMC4701921

[48] Pappalardo A Thompson K. Activated γ*δ* T cells inhibit osteoclast differentiation and resorptive activity *in vitro*. Clin Exp Immunol. (2013) 174:281–91. 10.1111/cei.12165 23815433 PMC3828832

[49] Yang G Chen X Yan Z Zhu Q Yang C. CD11b promotes the differentiation of osteoclasts induced by RANKL through the spleen tyrosine kinase signalling pathway. J Cell Mol Med. (2017) 21:3445–52. 10.1111/jcmm.13254 28661042 PMC5706498

[50] Salas A Sans M Soriano A Reverter JC Anderson DC Piqué JM. Heparin attenuates TNF-alpha induced inflammatory response through a CD11b dependent mechanism. Gut. (2000) 47:88–96. 10.1136/gut.47.1.88 10861269 PMC1727984

[51] Takeshita S Kaji K Kudo A. Identification and characterization of the new osteoclast progenitor with macrophage phenotypes being able to differentiate into mature osteoclasts. J Bone Miner Res. (2000) 15:1477–88. 10.1359/jbmr.2000.15.8.1477 10934646

[52] Petursdottir DH Hardardottir I. Dietary fish oil increases the number of splenic macrophages secreting TNF-alpha and IL-10 but decreases the secretion of these cytokines by splenic T cells from mice. J Nutr. (2007) 137:665–70. 10.1093/jn/137.3.665 17311957

[53] Cenci S Weitzmann MN Roggia C Namba N Novack D Woodring J. Estrogen deficiency induces bone loss by enhancing T-cell production of TNF-alpha. J Clin Invest. (2000) 106:1229–37. 10.1172/JCI11066 11086024 PMC381439

[54] Roggia C Gao Y Cenci S Weitzmann MN Toraldo G Isaia G. Up-regulation of TNF-producing T cells in the bone marrow: a key mechanism by which estrogen deficiency induces bone loss *in vivo*. Proc Natl Acad Sci USA. (2001) 98:13960–5. 10.1073/pnas.251534698 11717453 PMC61149

[55] Zhao W Liu Y Cahill CM Yang W Rogers JT Huang X. The role of T cells in osteoporosis, an update. Int J Clin Exp Pathol. (2009) 2:544–52. 19636401 PMC2713452

[56] Pacifici R. Role of T cells in ovariectomy induced bone loss–revisited. J Bone Miner Res. (2012) 27:231–9. 10.1002/jbmr.1500 22271394

[57] Poulsen RC Moughan PJ Kruger MC. Long-chain polyunsaturated fatty acids and the regulation of bone metabolism. Exp Biol Med. (2007) 232:1275–88. 10.3181/0704-MR-100 17959840

[58] Salari P Rezaie A Larijani B Abdollahi M. A systematic review of the impact of n-3 fatty acids in bone health and osteoporosis. Med Sci Monit. (2008) 14:RA37–44. 18301367

[59] Kelly OJ Gilman JC Kim Y Ilich JZ. Long-chain polyunsaturated fatty acids may mutually benefit both obesity and osteoporosis. Nutr Res. (2013) 33:521–33. 10.1016/j.nutres.2013.04.012 23827126

[60] Orchard TS Pan X Cheek F Ing SW Jackson RD. A systematic review of omega-3 fatty acids and osteoporosis. Br J Nutr. (2012) 107:S253–S260. 10.1017/S0007114512001638 22591899 PMC3899785

[61] Lavado-García J Roncero-Martin R Moran JM Pedrera-Canal M Aliaga I Leal-Hernandez O. Long-chain omega-3 polyunsaturated fatty acid dietary intake is positively associated with bone mineral density in normal and osteopenic Spanish women. PLoS ONE. (2018) 13:e0190539. 10.1371/journal.pone.0190539 29304057 PMC5755813

