# Supplementary figures and images for: Hexane Fraction of Turbo brunneus Inhibits Intermediates of RANK-RANKL Signaling Pathway and Prevent Ovariectomy Induced Bone Loss
Source: Front Endocrinol (Lausanne). 2019 Sep 6;10:608. doi: 10.3389/fendo.2019.00608 (PMC6742724; doi:10.3389/fendo.2019.00608)

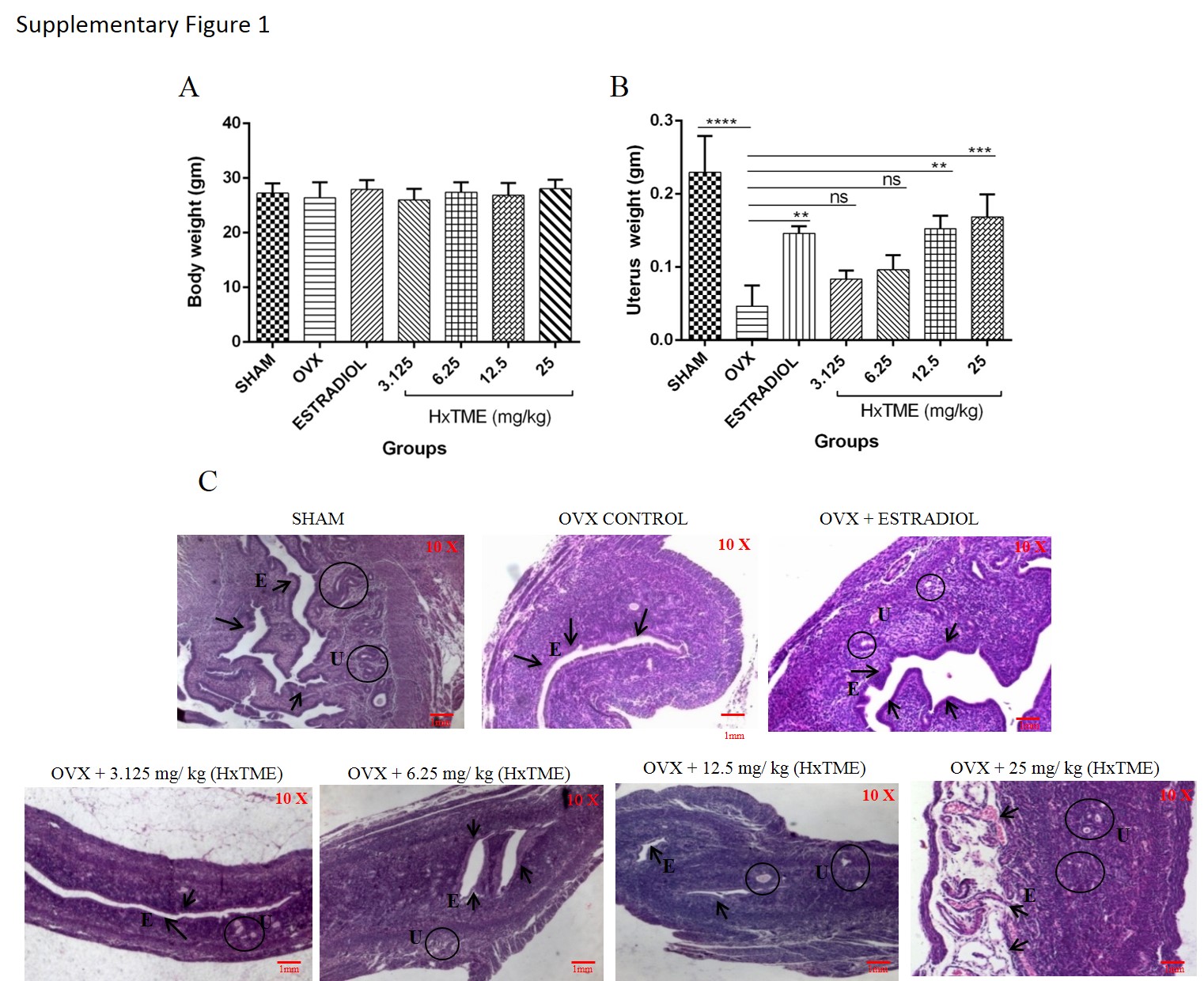

Supplement: Supplementary Figure 1 — Effect of HxTME fraction on body weight, uterine weight, and histology of ovariectomized mice upon HxTME treatment. After 5 weeks of oral administration of HxTME fraction, (A) body weight was measured in control, estradiol, and treated groups of ovariectomized mice. Similarly, (B) uterine weight was also measured after euthanization of control, estradiol, and treated groups of ovariectomized mice. All values are expressed as mean ± SEM, **P ≤ 0.01, ***P ≤ 0.001, and ****P ≤ 0.0001 vs. OVX. (C) Uterus was dissected out at the end of HxTME treatment. Uterus tissue was fixed in formalin, dehydrated in alcohol and paraffin sections were made. Sections were stained by using Haematoxylin and Eosin. Representative photomicrographs of uterine tissue were taken at 10X magnification. Scale bar = 1mm “E” (arrow) indicates uterine epithelium and “U” (circled) indicates uterine glands. [file Image_1.JPEG]

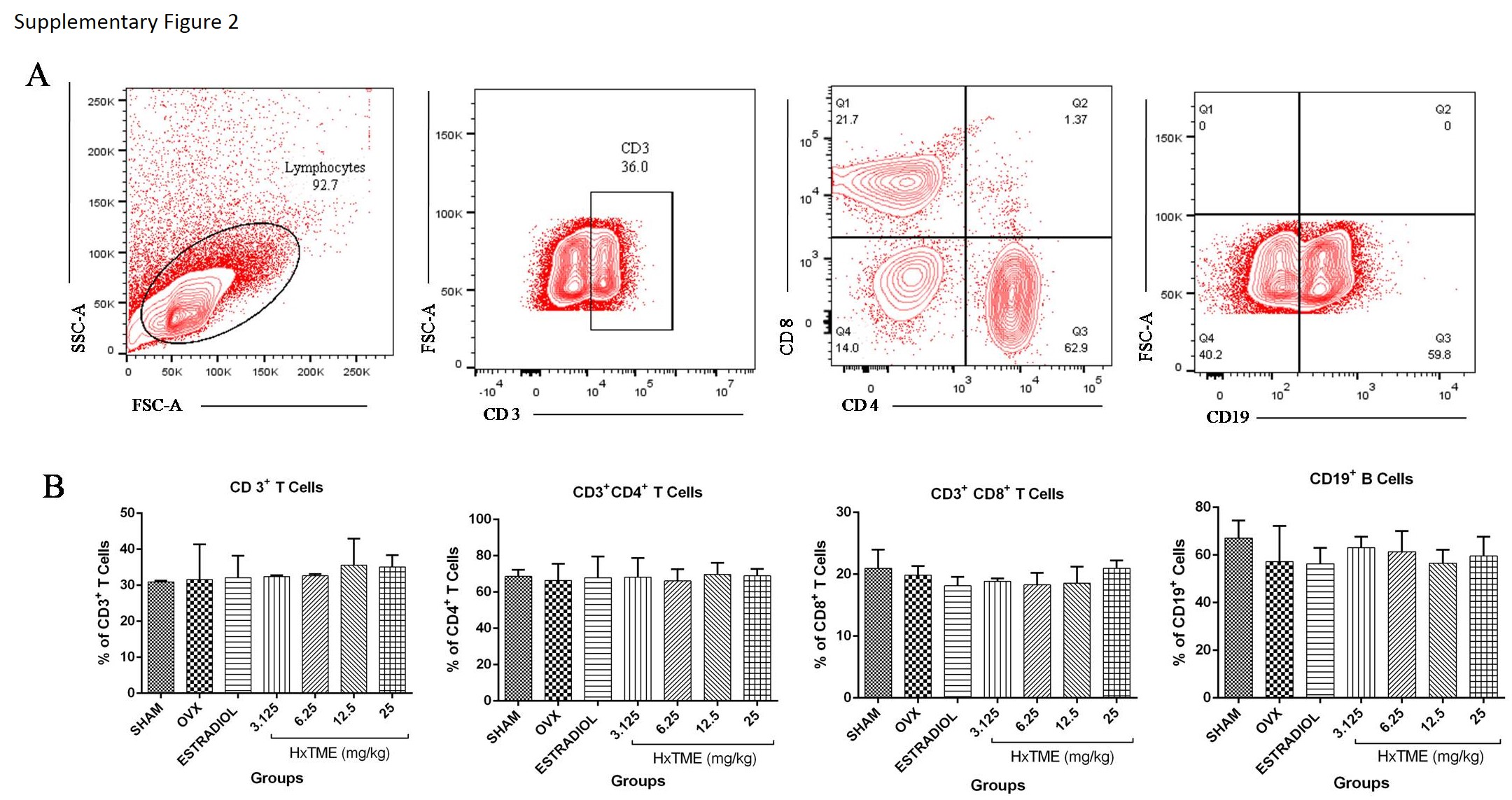

Supplement: Supplementary Figure 2 — Effect of HxTME on percentage population of T and B cell subsets in ovariectomized mice. (A) Gating strategies to analyse the percentage of T and B cell subsets in the splenocytes isolated from Sham, OVX, Estradiol, and HxTME treated OVX mice groups. (B) Bar plots show the percentage of total CD3+ T cells, CD3+CD4+ T cells, CD3+CD8+ T cells, and CD19+ cells. All values are expressed as mean. [file Image_2.JPEG]

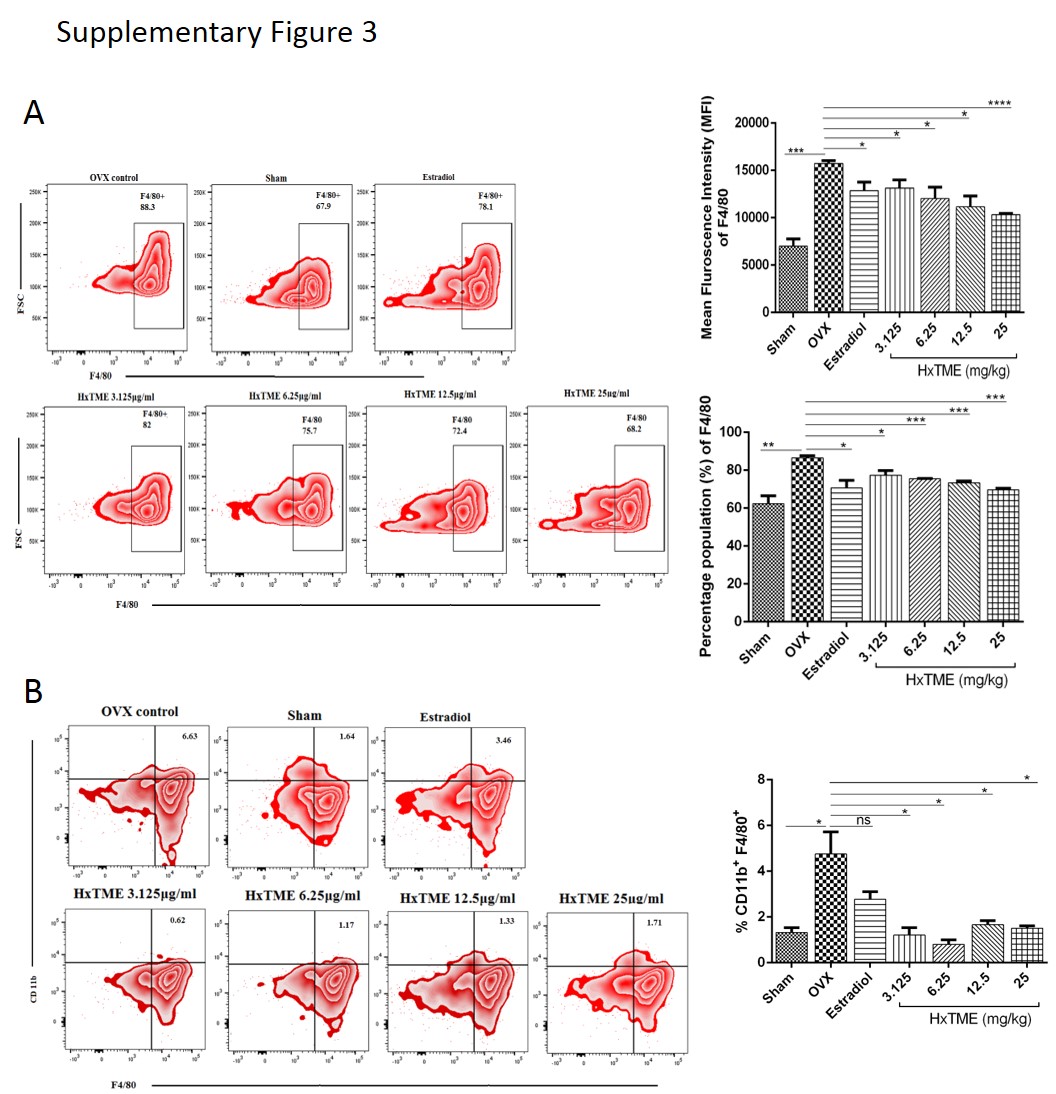

Supplement: Supplementary Figure 3 — Effect of HxTME on F4/80 osteoclast precursor marker. (A) Gating strategies and graphical representation (Bar Plots) of percentage and Mean Fluorescence Intensity of F4/80+ cells in the splenocytes isolated from Sham, OVX, Estradiol, and HxTME treated OVX mice groups. (B) Gating strategies and graphical representation (Bar Plots) of percentage and Mean Fluorescence Intensity of CD11b+ F4/80+ cells in the splenocytes isolated from Sham, OVX, Estradiol, and HxTME treated OVX mice groups. All values are expressed as mean ± SEM. *P ≤ 0.05, **P ≤ 0.01, ***P ≤ 0.001, and ****P ≤ 0.0001 vs. OVX. [file Image_3.JPEG]
